# NHC‐Copper(I)‐Mediated Functionalization of White Phosphorus

**DOI:** 10.1002/anie.8252739

**Published:** 2026-09-18

**Authors:** Thomas M. Horsley Downie, Sophia A. Vogler, Gábor Balázs, Francesco Cirigliano, Robert Wolf

**Affiliations:** ^1^ Institute of Inorganic Chemistry University of Regensburg Regensburg Germany

**Keywords:** copper, *N*‐heterocyclic carbene, phosphorus, polyphosphane, white phosphorus functionalization

## Abstract

We report a method for converting white phosphorus (P_4_) directly into tetra‐ and pentaphosphanes using *N*‐heterocyclic carbene (NHC) copper(I) complexes. The insertion of P_4_ into the metal‐element bonds of the NHC complexes (6Dipp)Cu(Nu) [Nu = O*t*Bu and N(*i*Pr)_2_] affords [(6Dipp)Cu(*κ* ^2^‐P_4_O*t*Bu)] (**1**) and [(6Dipp)Cu{*κ* ^2^‐P_4_N(*i*Pr)_2_}] (**3**), which exhibit rotation of the [P_4_Nu]^−^ ligand around the copper center in solution. This fluxional process is suppressed at low temperature, as shown by variable‐temperature ^31^P{^1^H} NMR spectroscopy. Treatment of **1** with W(CO)_5_(thf) resulted in the formation of the bimetallic complex [(6Dipp)Cu(μ,*κ* ^2^:*κ* ^1^‐P_4_O*t*Bu){W(CO)_5_}] (**2**) which does not show fluxional behavior. Reactions of **1** and **3** with p‐block element halides release metal‐free asymmetrically substituted bicyclo[1.1.0]tetraphosphabutane ‘butterfly’ compounds. The polyphosphanes P_4_(PMes_2_)(O*t*Bu) (**4a**), P_4_[P{N(*i*Pr)_2_}_2_](O*t*Bu) (**4b**), P_4_[P{N(*i*Pr)_2_}_2_]{N(*i*Pr)_2_} (**4c**), and P_4_(BPh_2_){N(*i*Pr)_2_} (**5**) were isolated as stable compounds. Treatment of **4c** with the NHC IMes induces further phosphorus–phosphorus bond cleavage and rearrangement to give NHC‐phosphinidene adduct [*cyclo*‐P_3_{N(*i*Pr)_2_}(P{N(*i*Pr)_2_}_2_)(P═IMes)] (**6**). Compound **4c** also reacts with W(CO)_5_(thf) and CuBr·SMe_2_ to give coordination complexes [W(CO)_5_{*κ* ^1^‐P_4_(P{N(*i*Pr)_2_}_2_)(N{*i*Pr}_2_)}] (**7**) and [Cu(Br){*κ* ^2^‐P_4_(P{N(*i*Pr)_2_}_2_)(N{*i*Pr}_2_)}] (**8**), respectively, demonstrating the ability of **4c** to serve as a mono‐ and bidentate ligand for transition metals. All compounds were characterized crystallographically and spectroscopically.

## Graphical abstract

White phosphorus is functionalized by *N*‐heterocyclic carbene supported copper(I) complexes through redox‐neutral steps. Treatment of the resulting complexes with electrophiles releases the ligands from the metal center as asymmetrically substituted organophosphorus butterfly compounds. These engage in monodentate or bidentate coordination chemistry, and treatment with an NHC induces rearrangement to an NHC‐phosphinidene adduct.

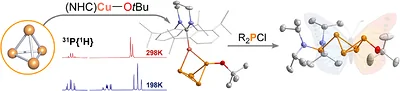

## 1 Introduction

The functionalization of white phosphorus (P_4_) has garnered significant attention due to its role as the primary industrial precursor for organophosphorus compounds, and the proclivity of P_4_ to yield diverse and unusual polyphosphorus complexes through metal‐mediated processes [1, 2, 3, 4, 5]. Activation of P_4_ by transition metal complexes frequently exploits low oxidation states to reduce the P_4_ tetrahedron, yielding aromatic polyphosphorus ligands such as [P_4_]^2−^ and [P_5_]^−^ [6, 7, 8, 9, 10]. While the reactivity of these ligands has been the focus of considerable attention, their notable stability makes them resistant to easy functionalization. Cleavage of an organophosphorus fragment from the metal center remains a challenge, requiring harsh reagents and conditions (Figure 1a) [7, 9, 11, 12, 13].

**FIGURE 1:**
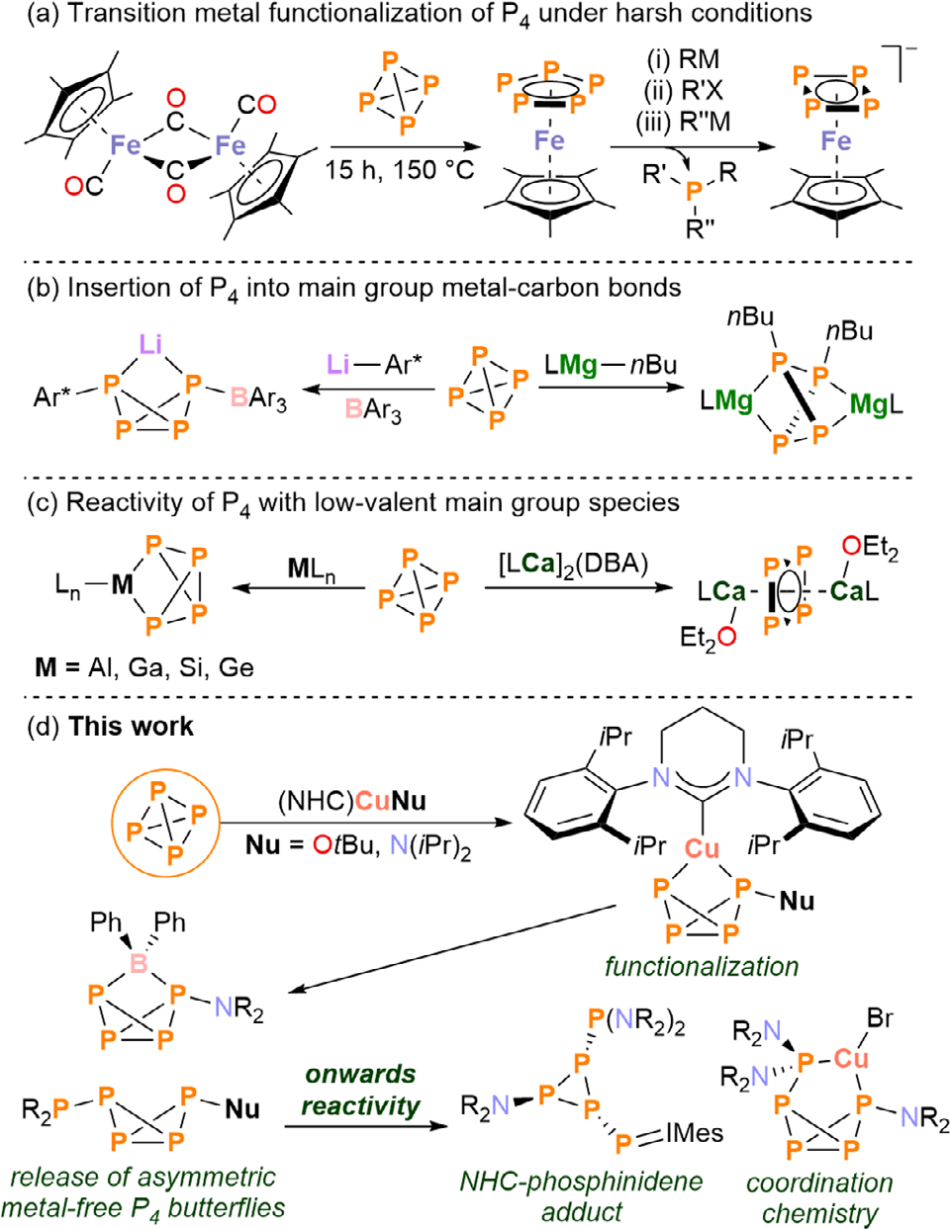
Selected examples of P_4_ functionalization (a) by transition metal complexes, (b) through insertion into metal–carbon bonds of main group complexes, (c) through activation by low‐valent main group species, and (d) through work presented in this study. Ar* = 2,4,6‐*t*Bu‐C_6_H_2_, 2,6‐Mes‐C_6_H_3_; Ar = C_6_F_5_; DBA = 9,10‐dimethyl‐diboraanthracene.

In contrast, P_4_ functionalization by main group species and lanthanides can occur through isohypsic steps [3, 14]. Treatment of P_4_ by aryl or alkyl complexes of lithium, gallium, magnesium and yttrium results, in each case, in the cleavage of one or more phosphorus–phosphorus bonds to insert the P_4_ molecule into the metal–carbon bonds (Figure 1b) [15, 16, 17, 18]. Lammertsma and colleagues showed that lithium bicyclo[1.1.0]tetraphosphabutanes, or phosphorus ‘butterfly’ complexes, can effectively be used to synthesize functionalized organophosphorus compounds [19, 20]. However, their application is constrained by the necessity for sterically bulky groups and potent Lewis acids to stabilize the [RP_4_]^−^ butterfly anions [16, 19].

In a related study, a mono‐substituted phosphorus ‘butterfly’ complex stabilized by two gold(I) centers was generated by the reaction between an *N*‐heterocyclic carbene (NHC) gold(I) compound and P_4_, followed by treatment with an aryl lithium reagent [21]. However, no further reaction chemistry was reported for this species as well as for related symmetrically‐substituted butterfly compounds, which were obtained by the addition aryl and cyclopentadienyl radicals to P_4_ using titanium(III) and copper(I) complexes [22, 23].

Isohypsic P_4_ functionalization using d‐block metal complexes is very uncommon. In addition to the previously mentioned NHC‐gold(I) mediated process, well‐documented examples are rare and appear to be limited to reactions of rhodium hydride, alkyl and aryl complexes [(triphos)MH_3_] (M = Rh, Ir) or [(triphos)Rh(R)(*η* ^2^‐C_2_H_4_)] (R = Me, Et, Ph; triphos = MeC(CH_2_PPh_2_)_3_) with P_4_, yielding [(triphos)M(*η* ^1^:*η* ^2^‐P_4_R)] compounds featuring a P─H or a P─C bond [24, 25].

In a different strategy, low oxidation state complexes of group 13/14 elements reduce P_4_ to give [P_4_]^2−^ butterfly complexes through oxidative addition [26, 27, 28, 29] and, more recently, strongly one‐electron reducing Mg(I) and Ca(I) complexes have been shown to give [P_4_]^2−^aromatic rings (Figure 1c) [30, 31]. However, similar to transition‐metal‐mediated P_4_ activation, this mimicry of transition metal behavior [32] usually stalls at the same fundamental problem: the generation of oligophosphorus complexes which have limited potential for further functionalization and especially for the ultimate release of the oligophosphorus moiety from the main‐group element template. Given the concomitant activation and functionalization of P_4_ offered by main group metal–carbon bonds, we were intrigued by the possibility of combining the reactivity profiles of both main group elements and transition metals.

Exploring this idea, we have studied the reactivity of P_4_ and other small molecules with heterobimetallic complexes that synergistically pair a transition metal with a main group or redox‐inactive *d*‐block metal. This approach has, thus far, examined Co–Mg [33], Co–Zn [34], Co–Ga [35, 36], Co–Si [37], Co–Sn [38, 39], Fe–Mg [40], Fe–Ga [36], and Fe–Sn [41] complexes, delivering diverse reactivity with P_4_ that has furnished us with multitudinous polyphosphorus complexes, for example, the inorganic sandwich complex [(*η* ^5^‐P_5_)Co(*η* ^3^‐P_3_)]^−^ [37] and the triple‐decker sandwich complex [(*t*Bu_2_C_2_P_2_)_2_Co_2_(*μ*‐P_4_)] [34, 39]. While these complexes revealed fundamental insights into the transformations of P_4_ in heterobinuclear coordination environments, the liberation of the oligophosphide ligand from the low‐oxidation state cobalt and iron centers expectedly proved difficult, showing that these systems were not suitable as templates for the preparation of metal‐free oligophosphorus species [13]. We sought, therefore, to combine properties of both main group and transition metals into a single, readily accessible monometallic complex.

Copper seemed ideally situated as a basis for such a complex, given the characteristics of copper(I) as a ‘pseudo main group’ element with low propensity for redox chemistry, but with a more benign reactivity profile than many reactive main group complexes that possess incompatibilities such as prohibitive oxophilicity. Specifically, we reasoned that NHC‐copper(I) alkoxide complexes, which are ubiquitous as pre‐catalysts in organic transformations [42, 43, 44], could serve as a widely available platform for P_4_ functionalization. Although the reaction between P_4_ and copper complexes has been documented, the P_4_ molecule has generally functioned merely as an intact, neutral entity [21, 45, 46, 47, 48, 49]. In this study, we use (NHC)‐copper(I) complexes to concurrently activate and functionalize white phosphorus in a redox‐inactive step. The resulting metal‐phosphorus complexes act as intermediates for further functionalization, from which metal‐free organophosphorus butterfly species can be isolated (Figure 1d). Additionally, as an initial evaluation of their role in the synthesis of further oligophosphanes, we describe our preliminary results on the reactivity of these butterfly compounds. Our results show that (NHC)‐copper(I) mediated P_4_ functionalization offers a direct and versatile route to novel metal‐free oligophosphorus compounds.

## 2 Results and Discussion

### 2.1 Synthesis and Characterization of NHC‐Copper(I)‐P_4_ Complexes

Addition of P_4_ to a solution of (6Dipp)CuO*t*Bu (6Dipp = 1,3‐bis(2,6‐diisopropylphenyl)‐3,4,5,6‐tetrahydropyrimidin‐2‐ylidene) in toluene immediately resulted in yellow coloration. [(6Dipp)Cu(*κ* ^2^‐P_4_O*t*Bu)] (**1**) gradually precipitated, allowing its isolation in an 87% yield (Scheme 1) [50]. When the reaction was carried out at a lower concentration, single crystals of 1 suitable for single‐crystal X‐ray diffraction (XRD) analysis formed in the reaction mixture overnight.

**SCHEME 1:**
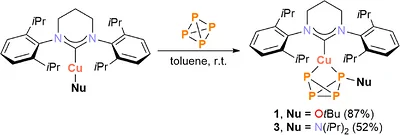
Synthesis of the NHC‐supported copper phosphide complexes **1** and **3**.

The solid‐state molecular structure of **1** (see Figure 2) reveals the insertion of P_4_ into the Cu─O bond of the copper alkoxide, with cleavage of one P─P bond of P_4_. While the *tert*‐butoxide group has been transferred to a phosphorus atom (P4), the copper center is now coordinated in a *κ* ^2^‐fashion to the [P_4_OR]^−^ ligand. The expected Cu1─P1 bond [2.3074(5) Å] is supplemented by a slightly longer dative coordination of the P4 atom to the metal center [2.3420(5) Å], leading the copper to sit astride the P_4_ ‘butterfly’ structure. Though the solid‐state structure of **1** would suggest three chemical environments for the phosphorus atoms, only two resonances are observed by ^31^P{^1^H} NMR spectroscopy at room temperature.

**FIGURE 2:**
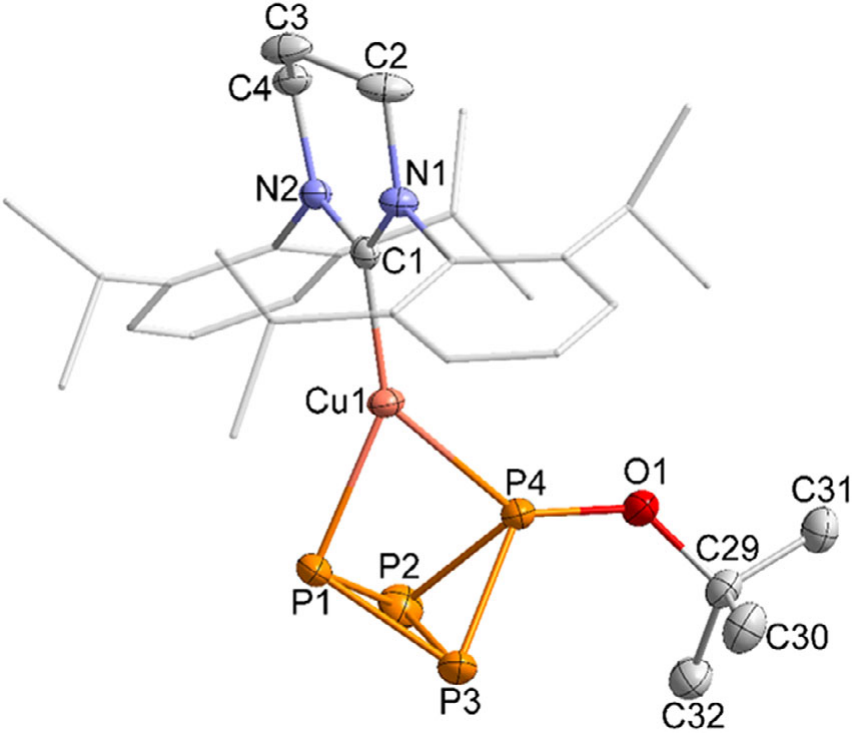
Solid‐state molecular structure of **1**. The hydrogen atoms are omitted and the diisopropylphenyl groups rendered in wireframe for clarity. Thermal ellipsoids are drawn at the 50% probability level. Selected bond lengths [Å] and angles [°]: Cu1‐C1 1.929(2), Cu1‐P1 2.3074(5), Cu1‐P4 2.3420(5), P1‐P2 2.1906(8), P1‐P3 2.1847(7), P2‐P3 2.1768(7), P2‐P4 2.2349(7), P3‐P4 2.2013(6); P1‐Cu1‐P4 72.32(2), Cu1‐P1‐P2 94.38(2), P1‐P2‐P4 76.61(2), and P1‐P2‐P3 60.03(2).

These appear to be an AX_3_ spin system, consisting of a quartet at *δ* −167 ppm and a doublet at *δ* −349 ppm. This is inconsistent with the solid‐state molecular structure, suggesting that **1** is subject to dynamic processes on an NMR timescale in solution, as previously observed for other P_4_ complexes [47, 48, 51, 52, 53]. We envision this as a perambulation of the copper center about the faces of a P_4_ tetrahedron with the alkoxide group affixed to one vertex, rendering the other three phosphorus atoms chemically equivalent on an NMR timescale.

This dynamic behavior is slowed at low temperature, as revealed by variable‐temperature (VT) ^31^P{^1^H} NMR spectra of **1** in thf‐*d*_8_, recorded at 20 K intervals down to 198 K (Figure 3). The resonance at *δ* −349 ppm broadens and then decoalesces below 258 K, gradually resolving into two new resonances at *δ* −341 and −352 ppm, with relative integrals of 1 and 2, respectively. This new AXY_2_ spin system corresponds closely to the resonances expected when considering the solid‐state structure of **1**. Iterative simulation of these higher‐order resonances provided reasonable *J*_PP_ coupling constants which, when fixed, allowed line‐shape analysis of the resonances at other temperatures (see Section SII). The derived rate constants *k* were used to estimate the Gibbs energy of activation for the dynamic exchange in solution at Δ*G* ^‡^_298K_ = 10.5 kcal·mol^−1^, comparable to other complexes exhibiting dynamic P_4_ exchange processes [47, 53]. Our postulated mechanism is further supported by density functional theory (DFT) calculations, which found a transition state in which the copper center passes over the face of an intact tetrahedron (see Section SV). The energetic barrier for rotation of the ligand derived from these calculations agrees closely with those established through line‐shape analysis (Δ*G* ^‡^_298K_ = 9.8 kcal·mol^−1^).

**FIGURE 3:**
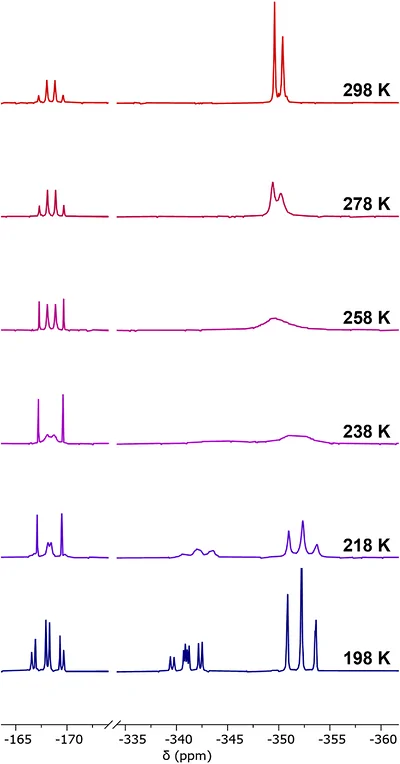
Variable‐temperature ^31^P{^1^H} NMR spectra of **1** in thf‐*d*_8_.

The dynamic behavior of **1** in solution can also be inhibited at room temperature by the presence of a Lewis acid to occupy the phosphorus atoms. Through treatment of **1** with W(CO)_5_(thf) in THF, followed by slow diffusion of hexane into the reaction mixture, [(6Dipp)Cu(*μ*,*κ* ^2^:*κ* ^1^‐P_4_O*t*Bu){W(CO)_5_}] (**2**) was isolated as orange crystals in a 72% yield. As observed in the crystal structure (Figure 4), the ‐W(CO)_5_ moiety is coordinated by the unoccupied lone pair of the wingtip phosphorus atom P1. Compound **2** thereby exhibits an AXY_2_ spin system in the ^31^P{^1^H} NMR spectrum (Figure S10) even at room temperature, illustrating that any solution exchange has been frustrated by locking the [P_4_OR]^−^ ligand into this ‘butterfly’ conformation. The preferential coordination to the wingtip reflects that this atom, bound to the metal center, is likely to be negatively charged relative to the more sterically accessible bridgehead phosphorus atoms, as calculated for germanium and zirconium P_4_ complexes [54, 55].

**FIGURE 4:**
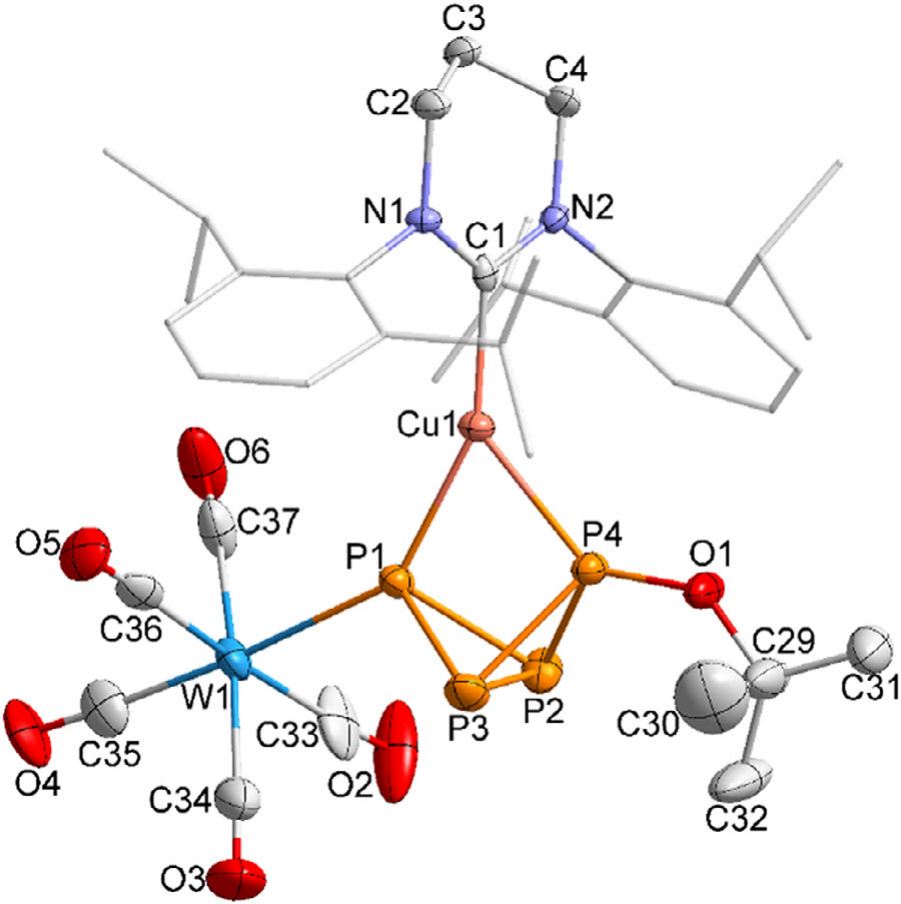
Solid‐state molecular structure of **2**. The hydrogen atoms are omitted and the diisopropylphenyl groups rendered in wireframe for clarity. Thermal ellipsoids are drawn at the 50% probability level. Selected bond lengths [Å] and angles [°]: Cu1‐C1 1.940(6), Cu1‐P1 2.296(2), Cu1‐P4 2.349(2), P1‐P2 2.209(3), P1‐P3 2.203(2), P2‐P3 2.184(3), P2‐P4 2.214(2), P3‐P4 2.218(2), W1‐P1 2.585(2); P1‐Cu1‐P4 71.69(6), Cu1‐P1‐P2 97.80(8), Cu1‐P1‐W1 144.07(8), P1‐P2‐P4 75.92(8), P1‐P2‐P3 60.21(8), and P2‐P1‐W1 111.11(8).

P_4_ failed to react with other (6Dipp)Cu–Nu complexes (where Nu = OPh, N(SiMe_3_)_2_, Mes), presumably as a result of lower nucleophilicity of these groups compared with the ‐O*t*Bu group. Thus, the diisopropylamide complex (6Dipp)CuN(*i*Pr)_2_ was synthesized due to the high basicity of such amides relative to their bis(trimethylsilyl) analogues. Gratifyingly, (6Dipp)CuN(*i*Pr)_2_ reacts with P_4_ in a manner akin to (6Dipp)CuO*t*Bu, giving [(6Dipp)Cu{*κ* ^2^‐P_4_N(*i*Pr)_2_}] (**3**) in a 52% yield (Scheme 1, vide supra). Compound **3** appears essentially isostructural to **1** in the solid state (Figure S89) and exhibits similar discrepancies between the solid and solution states as judged by ^31^P{^1^H} NMR spectroscopy (Figure S15). VT NMR of **3** shows that the two broad resonances at *δ* −121 and −317 ppm present at 298 K persist at elevated temperature with little resolution by 333 K (Figure S16). The resonances only decoalesce into three distinct peaks below 218 K, and unlike for **1**, they do not resolve again by 198 K, suggesting a lower energetic barrier for the rotation of the [P_4_NR_2_]^−^ ligand in solution.

The installation of alkoxide or amide groups onto a polyphosphorus framework derived from P_4_ is unusual. To the best of our knowledge, no examples with alkoxide groups are reported, and the only ‘butterfly’ compounds featuring amide groups are derived from the aggregation of P_2_ units, notably including the first phosphorus ‘butterfly’ species, P_4_{N(SiMe_3_)_2_}_2_, reported by Niecke and coworkers in 1982 [56, 57].

### 2.2 Synthesis of Asymmetric Metal‐Free Butterfly Compounds

Having characterized our NHC‐copper(I) P_4_ complexes, we sought to extrude a functionalized organophosphorus unit from the metal complex. Romerosa and co‐authors have noted in 2021 that while metal‐containing butterflies are reported through a variety of methods, ‘metal‐free analogues are still elusive and just a limited number of examples is described in the literature’ [58]. Moreover, most of these examples involve symmetrical substitution and include only a limited range of carbon‐centered substituents [59]. Thus, installing a second distinct functional group onto the P_4_^2−^ core would yield asymmetric ‘butterfly’ species which are vastly underreported compared to their symmetric counterparts, and with varied new motifs.

Treatment of **1** or **3** with a variety of halide‐containing electrophiles such as acyl chlorides or methyl iodide rapidly results in the formation of a precipitate, which could be identified as (6Dipp)CuX (X = Cl, I) crystallographically and spectroscopically. Analysis of the supernatant solutions by ^31^P{^1^H} NMR spectroscopy shows the formation of metal‐free ‘butterfly’ compounds with characteristic AMX_2_ spin systems, now cleaved from the copper center and functionalized with the corresponding electrophilic group (see Section SI.4 for in situ NMR spectroscopic analysis of these reactions). As Lammertsma and coworkers observed [16, 20], ‘butterfly’ species with insufficient steric support offered by the methyl group or an acyl moiety decompose rapidly. However, those substituted by a sufficiently bulky exocyclic phosphide group derived from treatment of **1** or **3** with a chlorophosphine are more robust and exhibit little degradation after prolonged storage. This is exemplified in the isolation of P_4_(PMes_2_)(O*t*Bu) (**4a**) from the reaction between **1** and Mes_2_PCl, in a yield of 59% (Figure 5a). The products are invariably highly soluble in hydrocarbon solvents and can simply be purified by extraction with pentane. In carrying out the synthesis of **4a**, the remaining precipitate (6Dipp)CuCl could be isolated from this reaction in a 77% yield and recycled for conversion back to the starting NHC‐copper(I) alkoxide.

**FIGURE 5:**
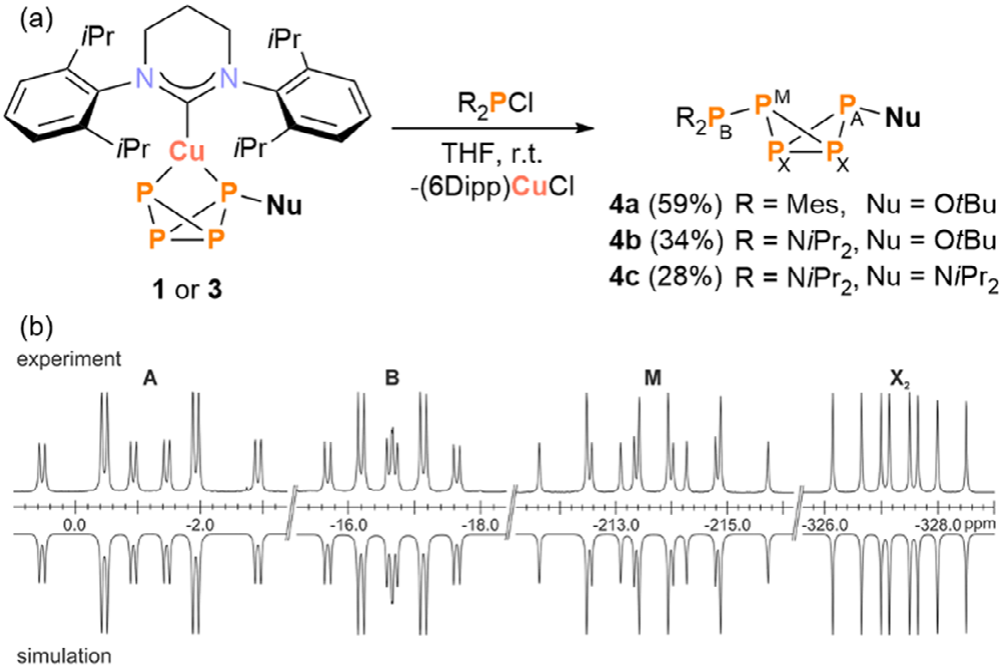
(a) Synthesis of metal‐free ‘butterfly’ compounds from complexes **1** and **3**. (b) Experimental (top) and simulated (bottom) ^31^P{^1^H} NMR spectra of compound **4a**: *δ*(P_A_) −1.2 ppm, *δ*(P_B_) −16.7 ppm, *δ*(P_M_) −213.7 ppm, *δ*(P_X_) −327.3 ppm; ^1^ *J*_AX_ = −201.9 Hz, ^1^ *J*_BM_ = −190.9 Hz, ^1^ *J*_MX_ = −172.5 Hz, ^2^ *J*_AM_ = 295.8 Hz, ^2^ *J*_BX_ = 102.4 Hz, and ^3^ *J*_AB_ = 18.5 Hz.

The ^31^P{^1^H} NMR spectrum of **4a** shows the expected ABMX_2_ spin system, and iterative simulation was used to obtain accurate coupling constants (Figure 5b). A high ^2^ *J*_PP_ coupling constant of 296 Hz between the wingtip phosphorus atoms denotes the orientation of their lone pairs toward one another [60], revealing the product is formed almost exclusively in an *exo*,*exo* configuration. The high solubility of the products impeded recrystallization of **4a**, though single crystals of the diamido‐substituted analogue P_4_[P{N(*i*Pr)_2_}_2_](O*t*Bu) (**4b**) were obtained, verifying that this *exo*,*exo* conformation persists in the solid state (Figure 6). The amido‐substituted ‘butterfly’ analogue P_4_[P{N(*i*Pr)_2_}_2_]{N(*i*Pr)_2_} (**4c**) is similarly synthesized from **3**, but the ^31^P{^1^H} NMR spectrum of the reaction reveals a mixture of *exo*,*exo* and *endo*,*exo* diastereomers in a 5:2 ratio (Figure S31). Nonetheless, the *exo*,*exo* butterfly crystallizes preferentially and was isolated in a 28% yield. NMR spectroscopic analysis of this material shows only pure *exo*,*exo*‐**4c**, demonstrating that the two diastereomers are not in equilibrium at room temperature. Thus, kinetic phenomena must be responsible for the generation of both in the reaction mixture, consistent with the typically high energy barrier for inversion at phosphorus centers. These compounds are isostructural in the solid‐state, though the greater steric encumbrance of the ‐N(*i*Pr)_2_ group of **4c** over the ‐O*t*Bu group of **4b** pushes it further out from the bridgehead P2/P3 atoms [(**4c**) P2‐P4‐N1 108.29(5)° vs. (**4b**) P2‐P4‐O1 99.38(7)°].

**FIGURE 6:**
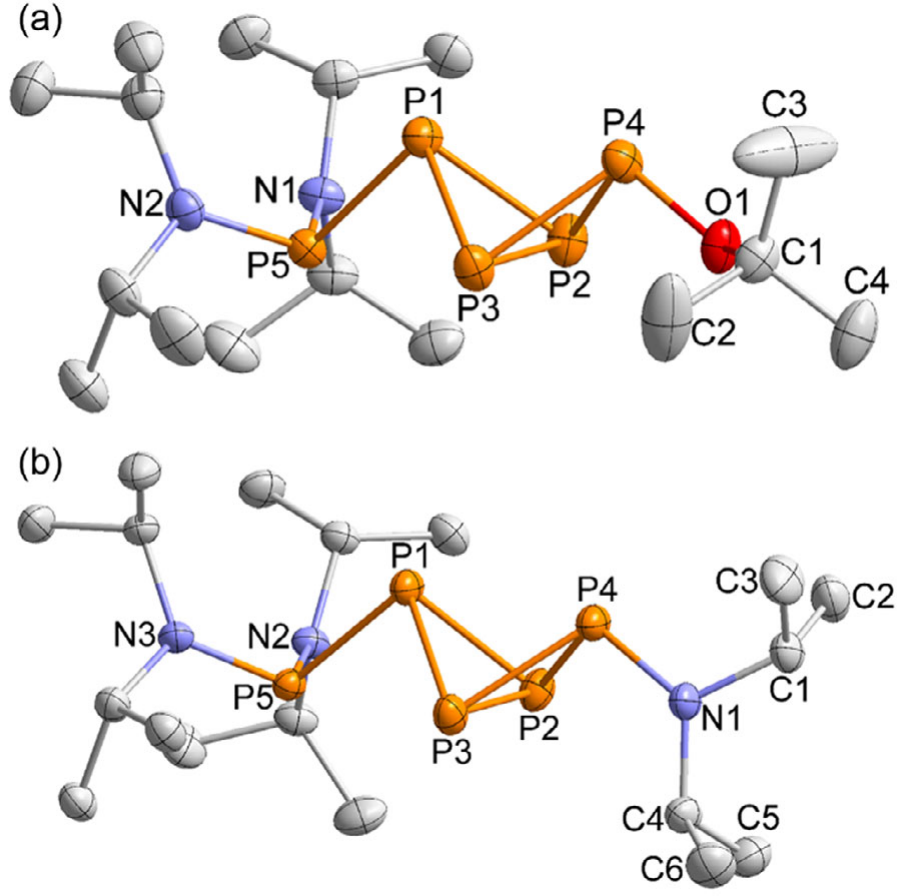
Solid‐state molecular structures of (a) **4b** and (b) **4c**. The hydrogen atoms are omitted for clarity. Thermal ellipsoids are drawn at the 50% probability level. Selected bond lengths [Å] and angles [°]: (**4b**) P1‐P2 2.2151(9), P2‐P3 2.1743(9), P2‐P4 2.2076(9), P1‐P5 2.2698(8), P4‐O1 1.6465(18); P1‐P2‐P3 60.51(3), P1‐P2‐P4 80.42(3), P2‐P1‐P5 93.52(3), P2‐P4‐O1 99.38(7); (**4c**) P1‐P2 2.2083(6), P2‐P3 2.1952(6), P2‐P4 2.2510(6), P1‐P5 2.2605(5), P4‐N1 1.6919(13); P1‐P2‐P3 60.459(19), P1‐P2‐P4 79.63(2), P2‐P1‐P5 94.11(2), and P2‐P4‐N1 108.29(5).

The reaction between **1** and other halide‐containing electrophiles, namely Me_3_SiCl and Ph_2_BCl, also resulted in the formation of (6Dipp)CuCl. However, the only phosphorus‐containing species detected by ^31^P{^1^H} NMR spectroscopy was regenerated P_4_. Due to the high oxophilicity of the silyl and boryl moieties, the rapid elimination of E–O*t*Bu is likely to be thermodynamically favourable upon formation of the P_4_(E)(O*t*Bu) (E = SiMe_3_, BPh_2_) species. In a similar reaction with **3**, the lack of the alkoxide group permits the isolation of a persistent metal‐free phosphorus product in the form of P_4_(BPh_2_){N(*i*Pr)_2_} (**5**) in a 53% yield (Scheme 2).

**SCHEME 2:**
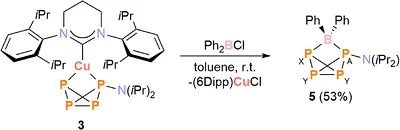
Synthesis of the boryl‐substituted butterfly compound **5**.

The ^2^ *J*_PP_ coupling constant between the wingtip phosphorus atoms indicates that unlike **4a**–**c**, compound **5** adopts an exclusively *endo*,*exo* conformation (Figure 7a). Furthermore, broadening of the P_A_ resonance at *δ* −88.1 ppm is characteristic of dative interaction of this phosphorus atom with the quadrupolar boron center. This was confirmed by single‐crystal XRD (Figure 7b), which revealed a tricyclic structure with the boryl moiety bridging the wingtip phosphorus atoms. While boron‐based Lewis acids have been frequently used by Lammertsma and coworkers to stabilize phosphorus ‘butterfly’ compounds [16, 19, 20], the incorporation of a boryl moiety as a substituent is precedented only by our report of P_4_(BPh_2_)(Fp*) (Fp* = (C_5_Me_5_)Fe(CO)_2_) earlier this year [40]. Notably, **5** constitutes an intramolecular Lewis pair with a purely inorganic backbone and, isolated here as a metal‐free compound, offers interesting potential for further reactivity as a Frustrated Lewis Pair [61] or ambiphilic ligand [62].

**FIGURE 7:**
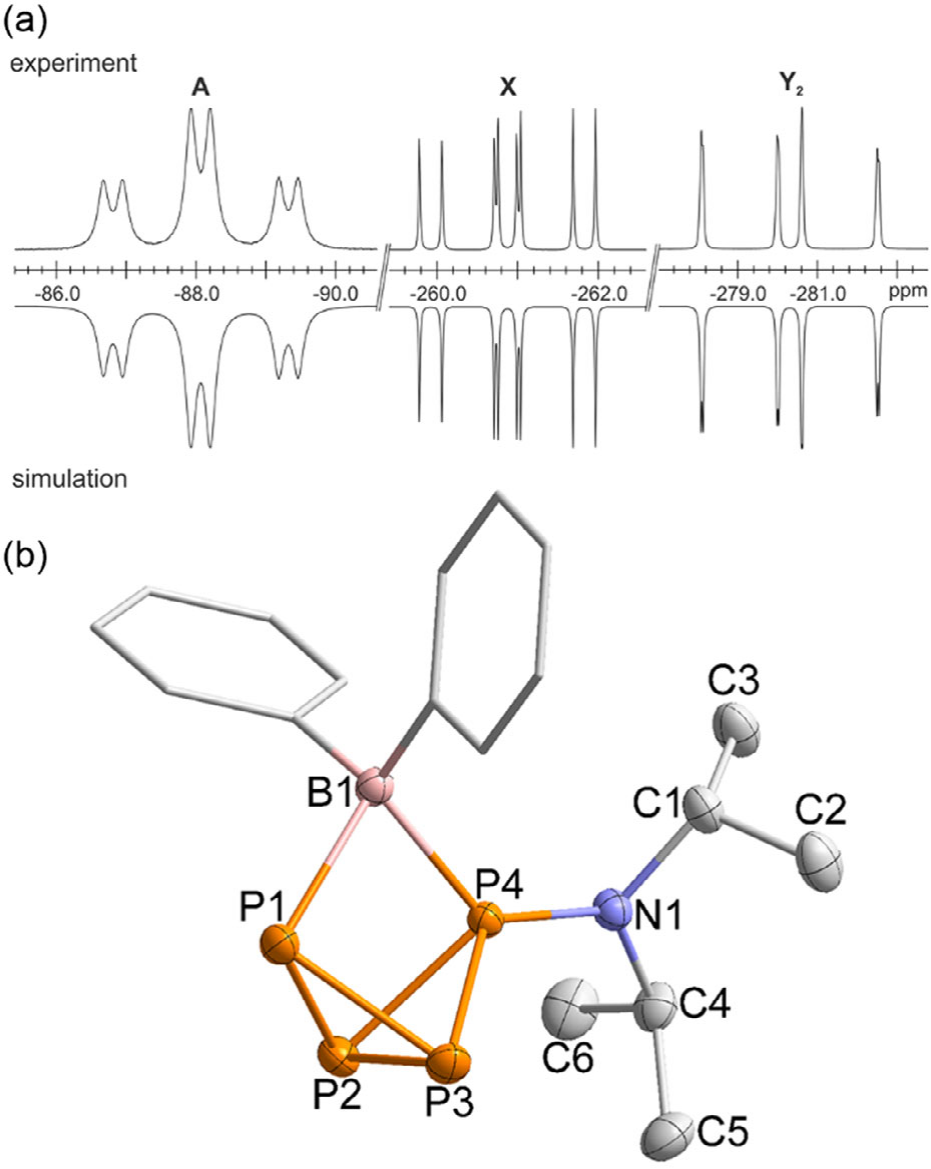
(a) Experimental (top) and simulated (bottom) ^31^P{^1^H} NMR spectra of compound **5**: *δ*(P_A_) = −88.1 ppm, *δ*(P_X_) = −260.9 ppm, *δ*(P_Y_) = −279.6 ppm; ^1^ *J*_AY_ = −254.4 Hz, ^1^ *J*_XY_ = −193.1 Hz, ^2^ *J*_AX_ = 56.3 Hz. (b) Solid‐state molecular structure of **5**. The hydrogen atoms are omitted and the phenyl groups rendered in wireframe for clarity. Thermal ellipsoids are drawn at the 50% probability level. Selected bond lengths [Å] and angles [°]: P1‐P2 2.2101(5), P2‐P3 2.2288(6), P2‐P4 2.1661(5), P1‐B1 2.070(2), P4‐B1 1.983(2), P4‐N1 1.649(1); P1‐P2‐P3 59.58(2), P1‐P2‐P4 73.16(2), B1‐P1‐P2 92.66(5), B1‐P4‐P2 96.47(5), P1‐B1‐P4 80.07(6), N1‐P4‐P2 122.63(5), and N1‐P4‐B1 130.59(6).

### 2.3 Reactivity of Metal‐Free Butterfly Compounds

Phosphorus butterfly species are proposed to represent the first intermediates in the breakdown of P_4_ to organophosphorus P_1_ compounds, both for the industrial synthesis of PX_3_ by treatment of P_4_ with halogens [63, 64] and for more recent routes adopting strategies such as radical chain reactions [22, 65], photoredox catalysis [66], and functionalization with disulfides [67]. Thus, having isolated examples of neutral, metal‐free butterfly compounds, we were surprised by the paucity of reactivity reported for such species. Having devised a strategy that enables us to access metal‐free butterfly compounds, we performed a reactivity study of **4c** as a representative compound.

Carbenes have been shown to react with P_4_ to give many different organophosphorus motifs depending on the electronics of the carbene [68, 69, 70, 71, 72], along with inducing cleavage of phosphorus‐phosphorus bonds in other polyphosphorus species [73, 74, 75]. Thus, **4c** was treated with two equivalents of IMes (1,3‐bis(2,4,6‐trimethylphenyl)imidazol‐2‐ylidene) to give one major product **6**. XRD analysis revealed the formation of a carbene‐phosphinidene substituted by a three‐membered phosphorus ring resulting from bond cleavage within the butterfly structure [76, 77]. The compound is presumably formed by attack of the NHC at the sterically accessible bridgehead phosphorus atom. This is followed by a rearrangement, which generates the three‐membered ring with the exocyclic NHC‐supported two‐coordinate phosphorus atom (Figure 8a).

**FIGURE 8:**
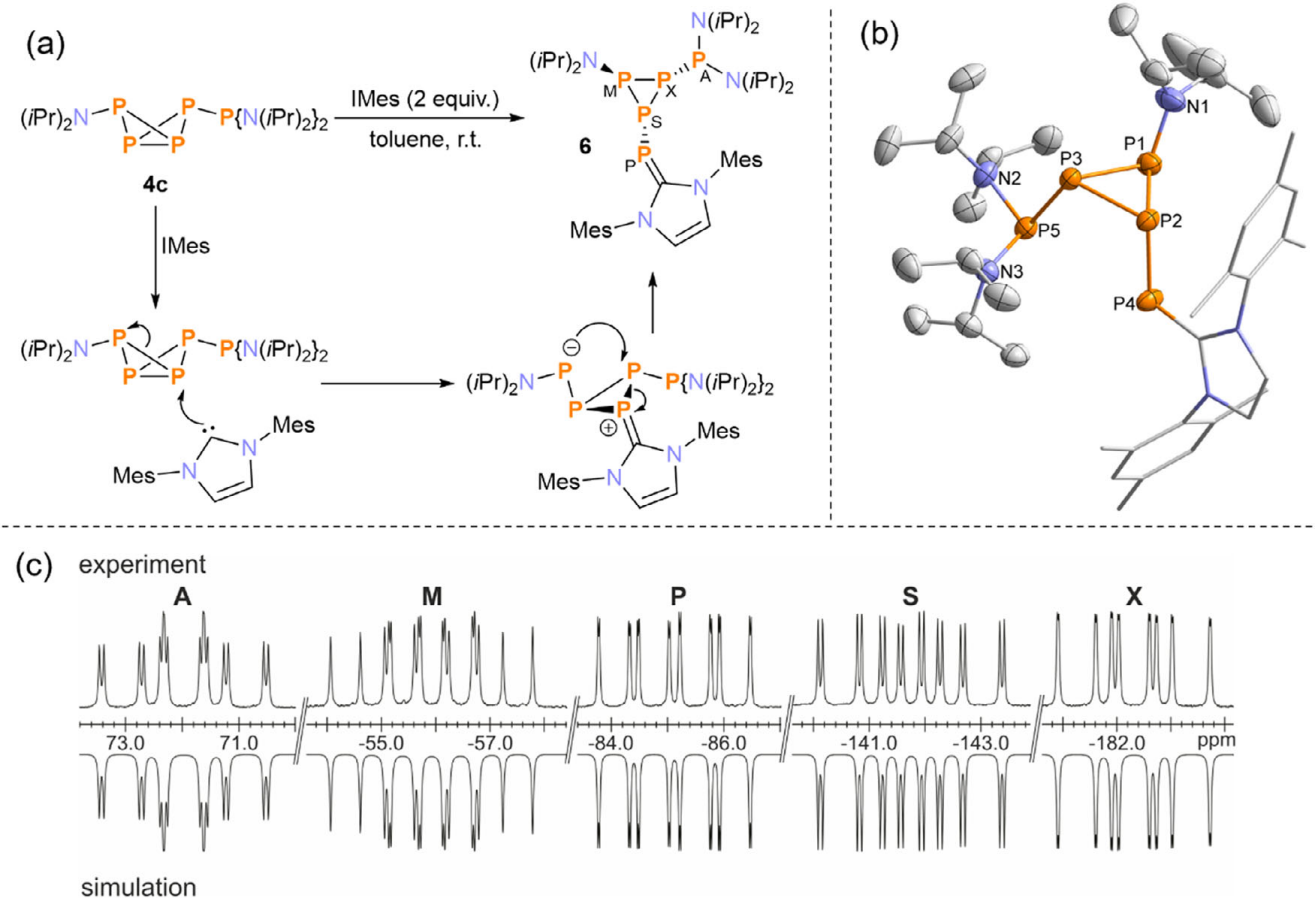
(a) Synthesis of the NHC‐phosphinidene adduct **6** via the proposed IMes‐induced rearrangement of **4c**. (b) The solid‐state molecular structure of **6**. Displacement ellipsoids are drawn at the 50% probability level. Hydrogen atoms have been omitted and IMes has been rendered in wireframe for clarity. Selected bond lengths [Å] and angles [°]: P1‐P2 2.2273(8), P1‐P3 2.1875(8), P2‐P3 2.2260(7), P2‐P4 2.1911(7), P3‐P5 2.2405(7), P4‐C19 1.773(2); P1‐P2‐P4 97.91(3), P1‐P3‐P5 99.09(3), N1‐P1‐P2 109.46(9), P3‐P2‐P4 104.05(3), P2 P3‐P5 113.34(3), P2‐P4‐C19 102.64(7). (c) Experimental (top) and simulated (bottom) ^31^P{^1^H} NMR spectra of compound **6**: *δ*(P_A_) = 72.0 ppm, *δ*(P_M_) = −56.0 ppm, *δ*(P_P_) = −85.1 ppm, *δ*(P_S_) = −141.7 ppm, *δ*(P_X_) = −182.3 ppm; ^1^ *J*_AX_ = −227.7 Hz, ^1^ *J*_MS_ = −225.2 Hz, ^1^ *J*_MX_ = −201.1 Hz, ^1^ *J*_PS_ = −291.7 Hz, ^1^ *J*_SX_ = −142.0 Hz, ^2^ *J*_AM_ = 216.3 Hz, ^2^ *J*_AS_ = 16.4 Hz, ^2^ *J*_MP_ = 110.8 Hz, ^2^ *J*_PX_ = 6.1 Hz, and ^3^ *J*_AP_ = 142.7 Hz.

Isolated samples of **6** showed contamination with varying amounts of IMes, owing to the similar solubilities of IMes and **6** (see Supporting Information for further details and spectroscopic data). Nonetheless, the constitution of **6** was unambiguously confirmed by single‐crystal XRD and multinuclear NMR spectroscopy. The solid‐state molecular structure of **6** shows a single diastereomer, with the exocyclic phosphido groups in a *cis* arrangement to each other and *trans* to the amido moiety (Figure 8b). Compound **6** gives rise to five multiplet resonances showing a dddd pattern in the ^31^P{^1^H} NMR spectrum between 80 and −190 ppm in the form of an AMPSX spin system (Figure 8c), reflecting the chemical inequivalence of the phosphorus atoms in the pentaphosphane. The relative loss of ring strain compared to the bicyclic butterfly structures is evident in the downfield chemical shifts in comparison with the resonances of **1–5**. The coupling constants, particularly the large ^3^ *J*_PP_ coupling of 142.7 Hz between the two exocyclic phosphorus atoms, indicate that the *cis* arrangement of the exocyclic phosphorus atoms is retained in solution. This stereochemistry presumably results from a kinetically controlled initial nucleophilic substitution step that occurs upon attack of the NHC as different P─P bonds are available for cleavage. Note that both in the reaction mixture or as an isolated compound, **6** is gradually converted to another oligophosphorus species assigned as an alternative diastereomer upon storage in solution for weeks (Figures S44 and 45). This observation is likely attributed to slow stereochemical inversion at phosphorus, which presumably results in a diastereomer with *trans*‐configured exocyclic phosphido groups. Compound **4c** also reacts with a less sterically‐hindered carbene to give P_1_ and P_2_ species, albeit with poor selectivity (see Section SI.5 for details).

Butterfly **4c** acts as a Lewis base and donor ligand with differing coordination chemistry for different metal centers in line with the literature. Treatment of **4c** with W(CO)_5_(thf) showed the clean formation of one new set of resonances retaining an AEMX_2_ spin system in the ^31^P{^1^H} NMR spectrum, with one resonance (P_E_, −26.5 ppm) featuring satellites resulting from *J*_WP_ coupling. This suggested a single favoured site of coordination for the tungsten center, confirmed by the isolation of tungsten complex [W(CO)_5_{*κ* ^1^‐P_4_(P{N(*i*Pr)_2_}_2_)(N{*i*Pr}_2_)}] (**7**) and analysis by single‐crystal XRD (Figure 9a). The solid‐state molecular structure shows coordination to the amido‐substituted wingtip phosphorus center, pointing to this as the center of highest Lewis basicity.

**FIGURE 9:**
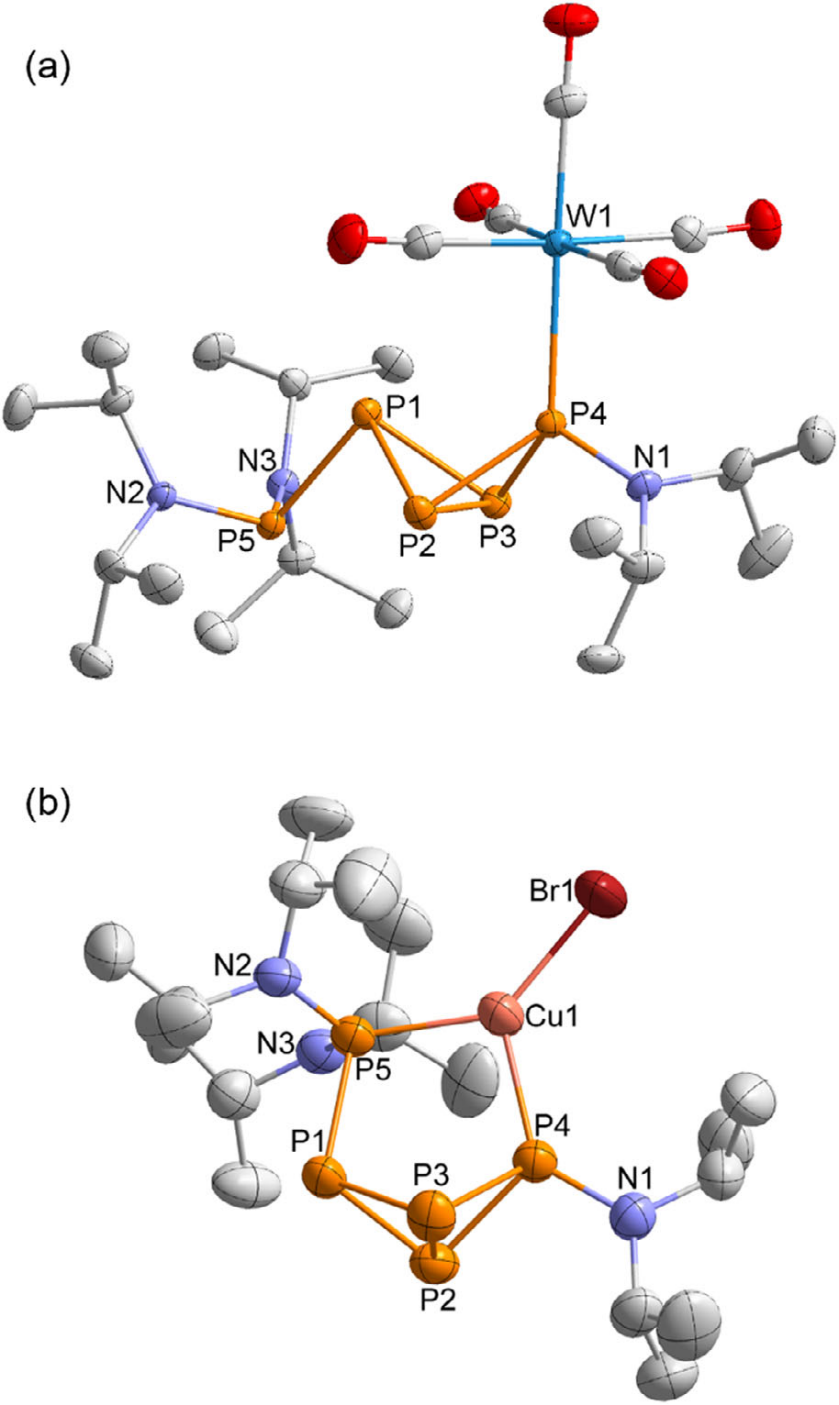
Solid‐state molecular structures of (a) **7** and (b) **8**. The hydrogen atoms are omitted for clarity. Thermal ellipsoids are drawn at the 50% probability level. Selected bond lengths [Å] and angles [°]: (**7**) W1‐P4 2.534(1), P1‐P5 2.271(2), P1‐P2 2.209(2), P2‐P4 2.212(2), P2‐P3 2.194(2); P1‐P2‐P4 83.03(7), P1‐P3‐P4 82.80(7), W1‐P4‐P2 125.60(7), P2‐P1‐P5 94.38(7); (**8**) Cu1‐P4 2.2677(9), Cu1‐P5 2.222(1), P1‐P2 2.175(1), P1‐P5 2.257(1), P2‐P4 2.232(1); P1‐P2‐P4 89.59(4), P5‐Cu1‐P4 96.38(3), P1‐P5‐Cu1 105.27(4), Cu1‐P4‐P2 114.76(4), and P2‐P1‐P5 106.15(5).

The reaction of **4c** with CuBr·SMe_2_ also gives rise to complex formation. Initial inspection of ^31^P{^1^H} NMR spectra revealed that both P_A_ and P_B_—at opposite ends of the oligophosphorus core—exhibited broadened resonances (at 30.6 and 7.5 ppm, respectively) indicative of coordination to a quadrupolar copper nucleus. XRD analysis revealed [Cu(Br){*κ* ^2^‐P_4_(P{N(*i*Pr)_2_}_2_)(N{*i*Pr}_2_)}] (**8**), in which a single copper center is chelated by the ligand, but in an unusual manner (Figure 9b). While the symmetrical iron‐substituted butterfly compound [{Cp′′′Fe(CO)_2_}_2_(*μ*,*η* ^1:1^‐P_4_)] (Cp′′′ = *η* ^5^‐C_5_H_2_ *t*Bu_3_) has been shown to act as a bidentate ligand with another metal center situated between the two wingtip atoms [78, 79, 80], in **8** the exocyclic ­P{N(*i*Pr)_2_}_2_ group acts as the additional donor, giving a five‐membered bicyclic chelate ring with the copper center. The complexation of CuBr to give **8** is slow and the product somewhat unstable, allowing for isolation of analytically pure material in only a low yield of 7%. Nonetheless, given sparse reports of related pentaphospanes in the literature [40, 81, 82], the synthesis of **8** highlights the intriguing potential of these butterfly compounds.

## 3 Conclusion

NHC‐copper(I) complexes (6Dipp)CuO*t*Bu and (6Dipp)CuN(*i*Pr)_2_ enable functionalization reactions of P_4_ through insertion into the Cu─O/Cu─N bond with concomitant P─P bond cleavage. The resulting copper complexes, **1** and **3**, bear functionalized [P_4_E]^−^ (*E* = O*t*Bu, N(*i*Pr)_2_) ligands which undergo dynamic rotation with respect to the metal center in solution at room temperature, as characterized by VT NMR, line‐shape analysis and DFT calculations. Treatment of **1** and **3** with electrophiles cleaves the ligand from the copper complex by salt metathesis to generate functionalized phosphorus butterfly species, bearing unusual asymmetric substitution patterns. We anticipate that simultaneous activation and functionalization of P_4_ using similar redox‐neutral strategies will provide a reliable and versatile means of generating new interesting oligophosphorus species under mild conditions. The reactivity of one such species, **4c**, with IMes, W(CO)_5_(thf), and CuBr·SMe_2_ resulted in the formation of NHC‐phosphinidene adduct **6** and coordination complexes **7** and **8**, respectively. These results illustrate the unexplored synthetic potential of these butterfly‐P_4_ intermediates, oft‐touted in the breakdown of P_4_ by main‐group or transition metal species. The constitution of **4a**–**c** renders them analogues of the ubiquitous diphosphine ligands such as dppe (1,2‐bis(diphenylphosphino)ethane) with an entirely inorganic backbone, and they feature inherent asymmetry and tunability which could be exploited for the development of homogeneous catalysts. Further coordination studies of such butterfly compounds, particularly with tuning of the substituents, will therefore be of considerable interest.

## Author Contributions

**Thomas M. Horsley Downie**: conceptualization, methodology, investigation, writing – original draft, writing – review and editing, and validation. **Sophia A. Vogler**: methodology, conceptualization, data curation, investigation, writing – original draft, writing – review and editing, and validation. **Gábor Balázs**: data curation, investigation, and writing – review and editing. **Francesco Cirigliano**: methodology and investigation. **Robert Wolf**: conceptualization, supervision, funding acquisition, writing – review and editing, project administration, and resources.

## Conflicts of Interest

The authors declare no conflicts of interest.

## Supporting information




**Supporting File**: anie74819‐sup‐0001‐SuppMat.docx.

## Data Availability

The data that supports the findings of this study are available in the Supporting Information of this article. The crystallographic data files are available through the CCDC [83].
