# Supplementary material for: NHC‐Copper(I)‐Mediated Functionalization of White Phosphorus
Source: Angew Chem Int Ed Engl. 2026 Sep 18;65(45):e8252739. doi: 10.1002/anie.8252739 (PMC13725586; doi:10.1002/anie.8252739)
Supplement: Supplementary file 1 [file ANIE-65-e8252739-s001.docx]

**Supporting Information**

**NHC-Copper(I) Mediated Functionalisation of White Phosphorus**

Thomas M. Horsley Downie,^+a^ Sophia A. Vogler,^+a^ Gábor Balázs,^a^ Francesco Cirigliano,^a^ and Robert Wolf*^a^

**Table of Contents**

[I.	Synthetic Procedures	1](#_Toc239151124)

[1	General Considerations	1](#_Toc239151125)

[2	Analytical Techniques	1](#_Toc239151126)

[3	Synthesis of Compounds	2](#_Toc239151127)

[4	NMR Scale Reactions	43](#_Toc239151128)

[5	Additional Experiments	55](#_Toc239151129)

[II.	Line-shape Analysis	60](#_Toc239151130)

[III.	Mass Spectra	62](#_Toc239151131)

[IV.	Single Crystal X-ray Diffraction Data	66](#_Toc239151132)

[V.	DFT Calculations	75](#_Toc239151133)

[VI.	References	83](#_Toc239151134)

# I. Synthetic Procedures

## 1 General Considerations

All reactions and manipulations were performed under an inert atmosphere of nitrogen using standard Schlenk-line or glovebox techniques (<0.1 ppm H_2_O and <0.1 ppm O_2_). All glassware was oven-dried at 160 °C overnight prior to use. Solvents were dried and degassed using a MBraun SPS800 solvent purification system. All dry solvents except *n*-hexane and *n*-pentane were stored under nitrogen or argon over activated 3 Å molecular sieves in gas-tight ampules. *n*-Hexane and *n*-pentane ^[1]^ were stored over a potassium mirror. *d_6_*-Benzene (C_6_D_6_) and *d_8_*-tetrahydrofuran (*d_8_*-THF) were dried over activated 3 Å molecular sieves.

6Dipp · HBF_4_,^[1]^ 6DippCuCl, (6Dipp)CuO*^t^*Bu,^[2]^ Mes_2_PCl^[3]^ and were prepared according to slightly modified literature procedures. [W(CO)_5_(thf)] was prepared by irradiation of a solution of [W(CO)_6_] in THF. All other chemicals were purchased from commercial suppliers. Liquids were purified by distillation prior to use. Commercial Cu(I)Cl was purified by dissolution in concentrated HCl, precipitation by dilution with water, washing of the solid with ethanol and diethyl ether and drying in vacuo.^[4]^ White phosphorus (P_4_) was purified by sublimation. All other chemicals were used as received.

## 2 Analytical Techniques

NMR spectra were recorded on Bruker Avance III HD 400 spectrometers or a Bruker Avance III HDX 500 MHz Ascend spectrometer at 300 K unless stated otherwise and internally referenced to residual solvent resonances (^1^H NMR: THF-d_8_: 1.72 ppm, C_6_D_6_: 7.16 ppm, ^13^C{^1^H} NMR: THF-d_8_: 25.31 ppm, C_6_D_6_: 128.06 ppm). Chemical shifts δ are given in [ppm] relative to external standards of tetramethyl silane (^1^H, ^13^C{^1^H}), 85% phosphorus acid (^31^P, ^31^P{^1^H}) and BF_3_·OEt_2_ (^11^B). The coupling constants *J* are given in [Hz]. Signals have been assigned by means of coupling constants, integral relations and 2D NMR spectroscopy (HMBC, HSQC, COSY).

For compounds exhibiting higher-order spin systems, the resolution enhanced ^31^P{^1^H} NMR spectra were processed using Cherwell Scientific's gNMR software.^[5]^ Full line-shape iteration was applied to obtain the best match between simulated and experimental spectra. ^1^*J*_PP_ coupling constants were attributed negative values, while the signs of other coupling constants were determined accordingly. The designation of the spin system was performed by convention.^[6]^

IR spectra were recorded on a Bruker ALPHA II Platinum ATR FT-IR spectrometer at room temperature. Elemental analyses and mass spectra were performed by the Central Analytical Department of the University of Regensburg.

## 3 Synthesis of Compounds

### Synthesis of (6Dipp)CuN(*^i^*Pr)_2_

A solution of potassium bis(trimethylsilyl)amide (2.13 g, 10.7 mmol, 1.05 equiv.) in THF (40 mL) was added to a solution of 6Dipp · HBF_4_ (5.0 g, 10 mmol, 1 equiv.) in THF (60 mL) to give a beige suspension. The suspension was stirred for 4 h, then filtered onto solid copper(I) chloride (1.06 g, 10.7 mmol, 1.05 equiv.). The mixture was stirred overnight in the absence of light. Volatiles were removed *in vacuo* to give a beige powder, which was washed with *n*‑hexane (2 × 40 mL) and dried. A solution of lithium diisopropylamide (1.14 g, 10.7 mmol, 1.05 equiv.) in THF (120 mL) was added to give a yellow-brown mixture which was stirred overnight. The solvent was removed *in vacuo*, the product was extracted in toluene and filtered through celite. After concentrating, the solution was stored at −30 °C overnight. The resulting crystalline material was isolated by filtration, washed with *n*-hexane (2 × 40 mL) and dried *in vacuo* to yield a neon-yellow solid. Suitable crystals for single crystal X-ray diffraction were obtained from a concentrated toluene solution of (6Dipp)CuN(*^i^*Pr)_2_ at −35 °C.

**Yield:** 1.96 g (3.44 mmol, 34%)

**^1^H NMR (400 MHz, C_6_D_6_, 300 K):** δ [ppm] = 0.77 (d, *J* = 6.2 Hz, 12H, N−CH(C*H*_3_)_2_), 1.21 (d, *J* = 6.9 Hz, 12H, CH(C*H*_3_)_2_), 1.52 (p, *J* = 5.9 Hz, 2H, CH_2_–C*H*_2_–CH_2_), 1.63 (d, *J* = 6.9 Hz, 12H, CH(C*H*_3_)_2_), 2.75 (t, *J* = 5.9 Hz, 4H, N–C*H*_2_), 3.10 (sept, *J* = 6.9 Hz, 4H, C*H*(CH_3_)_2_), 3.12 (sept, *J* = 6.2 Hz, 2H, N−C*H*(CH_3_)_2_), 7.09 – 7.12 (m, 4H, *m*-Ar–*H*), 7.20 - 7.24 (m, 2H, *p*-Ar–*H*).

**^13^C{^1^H} NMR (101 MHz, C_6_D_6_, 300 K):** δ [ppm] = 20.3 (CH_2_–*C*H_2_–CH_2_), 24.6 (CH(*C*H_3_)_2_), 24.9 (CH(*C*H_3_)_2_), 27.8 (N−CH(*C*H_3_)_2_), 28.6 (*C*H(CH_3_)_2_), 46.5 (N–*C*H_2_), 53.0 (N–CH(*C*H_3_)_2_), 124.7 (*m*-*C*–H, Ar), 128.7 (*p*-*C*–H, Ar), 142.7 (*i*-*C,* Ar), 145.5 (*o*-*C*, Ar), 203.5 (N−*C*−N).

**Elemental Analysis:** calcd. (%) for C_34_H_54_CuN_3_ (Mw = 568.37 g·mol^-1^): C 71.85, H 9.58, N 7.39; found C 71.97, H 9.56, N 6.97**.**

**Figure S1.** ^1^H NMR spectrum (400 MHz, C_6_D_6_, 300 K) of (6Dipp)CuN(*^i^*Pr)_2_; ● = toluene, * = C_6_D_6_.

**Figure S2.** ^13^C{^1^H} NMR spectrum (101 MHz, C_6_D_6_, 300 K) of (6Dipp)CuN(*^i^*Pr)_2_; * = C_6_D_6_.

### Synthesis of [(6Dipp)Cu(κ^2^-P_4_O*^t^*Bu)] (1)

A colourless solution of (6Dipp)CuO*^t^*Bu (514 mg, 0.95 mmol, 1 equiv.) in toluene (4 mL) was added to a colourless solution of P_4_ (130 mg, 1.05 mmol, 1.1 equiv.) in toluene (3 mL). The resulting yellow mixture was stirred for 1.5 hours, forming a pale yellow-beige suspension. The mixture was filtered and the precipitate was dried *in vacuo*, yielding [(6Dipp)Cu(κ^2^‑P_4_O*^t^*Bu)] as white powder. Suitable crystals for single-crystal X-ray diffraction were obtained by allowing a toluene solution of [(6Dipp)Cu(κ^2^‑P_4_O*^t^*Bu)] to stand overnight at ambient temperature.

**Yield:** 550 mg (0.83 mmol, 87%).

**^1^H NMR (400 MHz, THF-d_8_, 300 K):** δ [ppm] = 0.97 (s, 9H, O(C*H*_3_)_3_), 1.27 (d, *J* = 6.9 Hz, 12H, CH(C*H*_3_)_2_), 1.45 (d, *J* = 6.9 Hz, 12H, CH(C*H*_3_)_2_), 2.33 (p, *J* = 5.8 Hz, 2H, CH_2_–C*H*_2_–CH_2_), 3.16 (sept, *J* = 6.8 Hz, 4H, C*H*(CH_3_)_2_), 3.44 (t, *J* = 5.8 Hz, 4H, N–C*H*_2_), 7.21 – 7.23 (m, 4H, *m*-Ar–*H*), 7.30 – 7.33 (m, 2H, *p*-Ar–*H*).

**^13^C{^1^H} NMR (101 MHz, THF-d_8_, 300 K):** δ [ppm] = 20.2 (CH_2_–*C*H_2_–CH_2_), 24.1 (CH(*C*H_3_)_2_), 24.6 (CH(*C*H_3_)_2_), 28.5 (*C*H(CH_3_)_2_), 29.4 (O–C(*C*H_3_)_3_), 46.1 (N–*C*H_2_), 76.3 (d, ^2^*J*_CP_ = 13.9 Hz, O–*C*(CH_3_)_3_), 124.2 (*m*-*C*–H, Ar), 128.7 (*p*-*C*–H, Ar), 142.0 (*i*-*C,* Ar), 145.6 (*o*-*C*, Ar), 200.9 (N−*C*−N).

**^31^P{^1^H} NMR (162 MHz, THF-d_8_, 300 K):** δ [ppm] = −168.4 (q, ^1^*J*_PP_ = −130 Hz, 1P, P_A_), −350.0 (d, ^1^*J*_PP_ = −130 Hz, 3P, P_X_/P_Y_).

**^31^P{^1^H} NMR (162 MHz, THF-d_8_, 198 K):** δ [ppm] = −168.1 (m, ^1^*J*_AY_ = −226.1 Hz, ^2^*J*_AX_ = 60.9 Hz, 1P, P_A_), −340.9 (m, ^1^*J*_XY_ = −222.2 Hz, ^2^*J*_AX_ = 60.9 Hz, 1P, P_X_), −352.3 (m, ^1^*J*_AY_ = −226.1 Hz, ^1^*J*_XY_ = −222.2 Hz, 2P, P_Y_).

**Elemental Analysis:** calcd. (%) for C_32_H_49_CuN_2_OP_4_ (Mw = 665.20 g·mol^-1^) C 57.78, H 7.43, N 4.21; found C 57.94, H 7.56, N 4.19.

**Figure S3.** ^1^H NMR spectrum (400 MHz, THF-d_8_, 300 K) of [(6Dipp)Cu(κ^2^-P_4_O*^t^*Bu)] (**1**); * = THF-d_8_.

**Figure S4.** ^13^C{^1^H} NMR spectrum (101 MHz, THF-d_8_, 300 K) of [(6Dipp)Cu(κ^2^-P_4_O*^t^*Bu)] (**1**); * = THF-d_8_.

**Figure S5.** ^31^P{^1^H} NMR spectrum (162 MHz, THF-d_8_, 300 K) of [(6Dipp)Cu(κ^2^-P_4_O*^t^*Bu)] (**1**).

**Figure S6.** Variable-temperature ^31^P{^1^H} NMR spectra of [(6Dipp)Cu(κ^2^-P_4_O*^t^*Bu)] (**1**) in THF-d_8_ from 198 K to 298 K.

**Table S1.** Coupling constants from the iterative fit of the AXY_2_ spin system of [(6Dipp)Cu(κ^2^‑P_4_O*^t^*Bu)] (**1**) at 198 K; δ (P_A_) = −166.7 ppm, δ (P_X_) = −340.9 ppm, δ (P_Y_) = −353.3 ppm.

|  | ^1^*J*_AY_ = −226.1 Hz, ^1^*J_X_*_Y_ = −222.2 Hz, | *^2^J_AX_* = 60.9 Hz |
| --- | --- | --- |

**Figure S7.** Sections of the ^31^P{^1^H} NMR spectrum (162 MHz, THF-d_8_) of [(6Dipp)Cu(κ^2^-P_4_O*^t^*Bu)] (**1**) at 198 K; experiment (upwards) and simulation (downwards).

### Synthesis of [(6Dipp)Cu(µ,κ^2^:κ^1^-P_4_O*^t^*Bu){W(CO)_5_}] (2)

A colourless solution of [W(CO)_6_] (80.9 mg, 0.23 mmol, 1.4 equiv.) in THF (1 mL) was irradiated with UV-light (365 nm, 10 W) for 30 min while stirring. The solution turned deep yellow and was added to a solution of [(6Dipp)Cu(κ^2^-P_4_O*^t^*Bu)] (108 mg, 0.16 mmol, 1 equiv.) in THF. Slow diffusion of *n*-hexane into the solution at ambient temperature resulted in crystallisation of the product as orange blocks, which were dried *in vacuo* to give [(6Dipp)Cu(µ,κ^2^:κ^1^-P_4_O*^t^*Bu){W(CO)_5_}].

**Yield:** 114 mg (0.12 mmol, 72%).

**^1^H NMR (400 MHz, C_6_D_6_, 300 K):** δ [ppm] = 0.85 (s, 9H, O(C*H*_3_)_3_), 1.14 (d, *J* = 6.9 Hz, 12H, CH(C*H*_3_)_2_), 1.46 (p, *J* = 5.6 Hz, 2H, CH_2_–C*H*_2_–CH_2_), 1.55 (d, *J* = 6.9 Hz, 12H, CH(C*H*_3_)_2_), 2.75 (t, *J* = 5.9 Hz, 4H, N–C*H*_2_), 2.97 (sept, *J* = 6.8 Hz, 4H, C*H*(CH_3_)_2_), 7.10 – 7.12 (m, 4H, *m*-Ar–*H*), 7.18 – 7.22 (m, 2H, *p*-Ar–*H*).

**^13^C{^1^H} NMR (101 MHz, C_6_D_6_, 300 K):** δ [ppm] = 19.7 (CH_2_–*C*H_2_–CH_2_), 24.8 (CH(*C*H_3_)_2_), 24.9 (CH(*C*H_3_)_2_), 28.6 (*C*H(CH_3_)_2_), 29.3 (O−C(*C*H_3_)_3_), 46.9 (N–*C*H_2_), 124.8 (*m*-*C*–H, Ar), 129.5 (*p*-*C*–H, Ar), 141.3 (*i*-*C*, Ar), 145.2 (*o*-*C*–H, Ar), 198.5 (W−*C*O).

Signals corresponding to the carbene carbon and quaternary carbon of the *tert*-butoxide group were not detected.

**^31^P{^1^H} NMR (162 MHz, C_6_D_6_, 300 K):** δ [ppm] = −95.7 (dt, 1P, ^1^*J*_AY_ = −217.1 Hz, ^2^*J*_AX_ = 250.2 Hz), −310.4 (dt, 1P, ^1^*J*_XY_ = −184.7 Hz, ^2^*J*_AX_ = 250.2 Hz), −333.6 (dd, 2P, ^1^*J*_AY_ = −217.1 Hz, ^1^*J*_XY_ = −184.7 Hz).

**ATR-IR** (solid state)**:** ν [cm^-1^] = 2961 (m, CH), 2060 (m, CO), 1918 (s, CO), 1877 (s, CO).

**Elemental Analysis:** calcd. (%) for C_37_H_49_CuN_2_O_6_P_4_W (Mw = 990.1 g·mol^-1^): C 44.93, H 4.99, N 2.83; found C 45.08, H 5.06, N 2.82.

**Figure S8.** ^1^H NMR spectrum (400 MHz, C_6_D_6_, 300 K) of [(6Dipp)Cu(µ,κ^2^:κ^1^-P_4_O*^t^*Bu){W(CO)_5_}] (**2**); * = C_6_D_6_.

**Figure S9**. ^13^C{^1^H} NMR spectrum (101 MHz, C_6_D_6_, 300 K) of [(6Dipp)Cu(µ,κ^2^:κ^1^-P_4_O*^t^*Bu){W(CO)_5_}] (**2**); * = C_6_D_6_.

**Figure S10.** ^31^P{^1^H} NMR spectrum (162 MHz, C_6_D_6_, 300 K) of [(6Dipp)Cu(µ,κ^2^:κ^1^-P_4_O*^t^*Bu){W(CO)_5_}] (**2**); * = P_4_.

**Table S2.** Coupling constants from the iterative fit of the AXY_2_ spin system of [(6Dipp)Cu(µ,κ^2^:κ^1^-P_4_O*^t^*Bu){W(CO)_5_}] (**2**); δ (P_A_) = −95.7 ppm, δ (P_X_) = −310.4 ppm, δ (P_Y_) = −333.6 ppm.

|  | ^1^*J*_AY_ = −217.1 Hz ^1^*J_X_*_Y_ = −184.7 Hz | *^2^J_AX_* = 250.2 Hz |
| --- | --- | --- |

**Figure S11.** Sections of the ^31^P{^1^H} NMR spectrum (162.0 MHz, C6D6, 300 K) of [(6Dipp)Cu(µ,κ^2^:κ^1^-P_4_O*^t^*Bu){W(CO)_5_}] (**2**); experiment (upwards) and simulation (downwards).

**Figure S12.** IR spectrum (300 K) of [(6Dipp)Cu(µ,κ^2^:κ^1^-P_4_O*^t^*Bu){W(CO)_5_}] (**2**).

### Synthesis of [(6Dipp)Cu{κ^2^-P_4_N(*^i^*Pr)_2_}] (3)

A colourless solution of P_4_ (61.9 mg, 0.50 mmol, 1 equiv.) in toluene (2 mL) was added to a yellow solution of (6Dipp)CuN(*^i^*Pr)_2_ (284 mg, 0.50 mmol, 1 equiv.) in toluene (2.5 mL). The resulting orange solution was stirred for 3 hours, forming an orange suspension. The mixture was filtered and the precipitate was dried *in vacuo*, yielding [(6Dipp)Cu{κ^2^‑P_4_N)*^i^*Pr_2_)}] as beige powder. Suitable crystals for single-crystal X‑ray diffraction were obtained from a concentrated toluene solution of [(6Dipp)Cu{κ^2^‑P_4_N(*^i^*Pr)_2_}] at −35 °C. The compound undergoes slow decomposition at 60 °C or upon prolonged storage in solution.

**Yield:** 180 mg (0.26 mmol, 52%)

**^1^H NMR (500 MHz, THF-d_8_, 300 K):** δ [ppm] = 0.88 (d, *J* = 6.8 Hz, 12H, N−CH(C*H*_3_)_2_), 1.28 (d, *J* = 6.9 Hz, 12H, CH(C*H*_3_)_2_), 1.47 (d, *J* = 6.9 Hz, 12H, CH(C*H*_3_)_2_), 2.33 (p, *J* = 5.8 Hz, 2H, CH_2_–C*H*_2_–CH_2_), 2.86 (m, overlapping sept, *J* = 6.8 Hz, 2H, N−C*H*(CH_3_)_2_), 3.18 (sept, *J* = 6.9 Hz, 4H, C*H*(CH_3_)_2_), 3.42 (t, *J* = 5.8 Hz, 4H, N–C*H*_2_), 7.22 – 7.24 (m, 4H, *m*-Ar–*H*), 7.29 – 7.33 (m, 2H, *p*-Ar–*H*).

**^13^C{^1^H} NMR (126 MHz, THF-d_8_, 300 K):** δ [ppm] = 21.4 (CH_2_–*C*H_2_–CH_2_), 23.8 (N–CH(*C*H_3_)_2_), 25.4 (CH(*C*H_3_)_2_), 29.4 (*C*H(CH_3_)_2_), 47.4 (N–*C*H_2_), 47.9 (N–*C*H(CH_3_)_2_), 125.2 (*m*-*C*–H, Ar), 129.5 (*p*-*C*–H, Ar), 143.1 (*i*-*C,* Ar), 146.5 (*o*-*C*, Ar), 202.3 (N−*C*−N).

**^31^P{^1^H} NMR (202 MHz, THF-d_8_, 300 K):** δ [ppm] = −120.8 (br s, 1P, P_A_), −317.1 (br s, 3P, P_X_/P_Y_).

**^31^P{^1^H} NMR (162 MHz, THF-d_8_, 198 K):** δ [ppm] = −124.8 (q, 1P, P_A_), −279.5 (br s, 1P, P_X_), −327.2 (br s, 2P, P_Y_).

**Elemental Analysis:** calcd. (%) for C_34_H_54_CuN_3_P_4_ (Mw = 692.27 g·mol^-1^): C 58.99, H 7.86, N 6.07; found C 58.54, H 7.61, N 5.77.

**Figure S13.** ^1^H NMR spectrum (500 MHz, THF-d_8_, 300 K) of [(6Dipp)Cu{κ^2^-P_4_N(*^i^*Pr)_2_}] (**3**); * = THF-d_8_.

**Figure S14.** ^13^C{^1^H} NMR spectrum (126 MHz, THF-d_8_, 300 K) of [(6Dipp)Cu{κ^2^-P_4_N(*^i^*Pr)_2_}] (**3**); * = THF-d_8_.

**Figure S15.** ^31^P{^1^H} NMR spectrum (202 MHz, THF-d_8_, 300 K) of [(6Dipp)Cu{κ^2^-P_4_N(*^i^*Pr)_2_}] (**3**).

**Figure S16.** Variable-temperature ^31^P{^1^H} NMR spectra of [(6Dipp)Cu{κ^2^-P_4_N(*^i^*Pr)_2_}] (**3**) in THF-d_8_ from 198 K to 333 K.

### Synthesis of *exo,exo*-P_4_(PMes_2_)(O*^t^*Bu) (4a)

A solution of [(6Dipp)Cu(κ^2^-P_4_O*^t^*Bu)] (206 mg, 0.31 mmol, 1.1 equiv.) in THF (4 mL) was added to a colourless solution of bis(2,4,6-trimethylphenyl)phosphorus chloride (86 mg, 0.28 mmol, 1 equiv.) in THF (1 mL). The reaction mixture was stirred for 1 hour, after which precipitate was observed. The solvent was removed *in vacuo*, and the product was extracted in *n*-pentane, followed by stirring the mixture overnight. The solution was filtered and the solvent of the filtrate was removed *in vacuo*. The product was isolated as a yellow solid.

**Yield:** 77.9 mg (0.17 mmol, 59%)

**^1^H NMR (500 MHz, C_6_D_6_, 300 K):** δ [ppm] = 0.99 (s, 9H, OC(C*H*_3_)_3_), 2.03 (s, 6H, *p*‑C*H*_3_), 2.48 (s, 12H, *o*-C*H*_3_), 6.61 – 6.63 (m, 4H, Ar–*H*).

**^13^C{^1^H} NMR (126 MHz, C_6_D_6_, 300 K):** δ [ppm] = 20.5 (s, p-*C*H_3_, Ar), 24.0 (dd, *J* = 8.3 Hz, *J* = 11.8 Hz, o-*C*H_3_, Ar), 29.0 (d, *J* = 7.0 Hz, OC(*C*H_3_)_3_), 78.2 (s, O*C*(CH_3_)_3_), 130.2 (d, *J* = 2.0 Hz, *m*-*C*, Ar), 133.7 (m, *o*-*C*, Ar), 137.9 (s, *p*-*C*, Ar), 141.5 (d, *J* = 14.4 Hz, *i*-*C*, Ar).

**^31^P{^1^H} NMR (202 MHz, C_6_D_6_, 300 K):** δ [ppm] = −1.2 (dtd, ^1^*J*_AX_ = −201.9 Hz, ^2^*J*_AM_ = 295.8 Hz, ^3^*J*_AB_ = 18.5 Hz, 1P, P_A_), −16.7 (dtd, ^1^*J*_BM_ = −190.9 Hz, ^2^*J*_BX_ = 102.4 Hz, ^3^*J*_AB_ = 18.5 Hz, 1P, P_B_), −213.7 (ddt, ^1^*J*_BM_ = −190.9 Hz, ^1^*J*_MX_ = −172.5 Hz, ^2^*J*_AM_ = 295.8 Hz, 1P, P_M_), −327.3 (ddd, ^1^*J*_AX_ = −201.9 Hz, ^1^*J*_MX_ = −172.5 Hz, ^2^*J*_BX_ = 102.4 Hz, 2P, P_X_).

**Elemental Analysis:** calcd. (%) for C_22_H_31_OP_5_ (Mw = 466.36 g·mol^-1^): C 56.66, H 6.70; found C 58.64, H 6.41

Despite repeated attempts, satisfactory elemental analysis could not be obtained. NMR spectroscopic analysis revealed the presence of Mes₂PH (0.14 equiv.), P₄ (0.03 equiv.), (Mes_2_P)_2_ (0.01 equiv.) and minor unidentified impurities. Complete purification was impeded by the similar solubilities of the product and impurities.

**FD-MS (*n*-hexane) m/z (%):** **466.1084 (100) for [M^+^]**.

**Figure S17.** ^1^H NMR spectrum (500 MHz, C_6_D_6_, 300 K) of *exo,exo*-P_4_(PMes_2_)(O*^t^*Bu) (**4a**); **○** = Mes_2_PH, * = C_6_D_6_.

**Figure S18.** ^13^C{^1^H} NMR spectrum (126 MHz, C_6_D_6_, 300 K) of *exo,exo*-P_4_(PMes_2_)(O*^t^*Bu) (**4a**), **○** = Mes_2_PH, * = C_6_D_6_**.**

**Figure S19.** ^31^P{^1^H} NMR spectrum (162 MHz, C_6_D_6_, 300 K) of *exo,exo*-P_4_(PMes_2_)(O*^t^*Bu) (**4a**), **○** = Mes_2_PH, * = P_4_.

**Table S3.** Coupling constants from the iterative fit of the ABMX_2_ spin system of *exo,exo*-P_4_(PMes_2_)(O*^t^*Bu) (**4a**); δ (P_A_) = −1.2 ppm, δ (P_B_) = −16.7 ppm, δ (P_M_) = −213.7 ppm, δ (P_X_) = −327.3 ppm.

|  | ^1^*J*_AX_ = −201.9 Hz  ^1^*J*_BM_ = −190.9 Hz ^1^*J*_MX_ = −172.5 Hz | ^2^*J*_AM_ = 295.8 Hz  ^2^*J*_BX_ = 102.4 Hz | ^3^*J*_AB_ = 18.5 Hz |
| --- | --- | --- | --- |

**Figure S20.** Sections of the ^31^P{^1^H} NMR spectrum (202.0 MHz, C_6_D_6_, 300 K) of *exo,exo*-P_4_(PMes_2_)(O*^t^*Bu) (**4a**); experiment (upwards) and simulation (downwards).

### Synthesis of *exo,exo*-P_4_[P{N(*^i^*Pr)_2_}_2_](O*^t^*Bu) (4b)

A solution of [(6Dipp)Cu(κ^2^-P_4_O*^t^*Bu)] (**1**) (333 mg, 0.50 mmol, 1 equiv.) in THF (4 mL) was added to a colourless solution of bis(diisopropylamino)phosphorus chloride (133 mg, 0.50 mmol, 1 equiv.) in THF (2 mL). The resulting light yellow reaction mixture was stirred at room temperature for three days, during which a precipitate formed. The solvent was removed *in vacuo* and the product was extracted with *n*-pentane (3 × 3 mL). The combined extracts were filtered, concentrated and stored at −35 °C, resulting in colourless crystals. The supernatant solution was decanted and the product was dried *in vacuo*. Recrystallisation from *n*-pentane at −35 °C afforded colourless blocks of *exo,exo*-P_4_[P{N(*^i^*Pr)_2_}_2_](O*^t^*Bu).

**Yield:** 72 mg (0.17 mmol, 34%)

**^1^H NMR (500 MHz, C_6_D_6_, 300 K):** δ [ppm] = 1.05 (d, *J* = 6.7 Hz, 12H, NCH(C*H*_3_)_2_), 1.08 (s, 9H, OC(C*H*_3_)_3_), 1.23 (d, *J* = 6.7 Hz, 12H, NCH(C*H*_3_)_2_), 3.41 (sept, *J* = 6.7 Hz, 2H, NC*H*(CH_3_)_2_).

**^13^C{^1^H} NMR (126 MHz, C_6_D_6_, 300 K):** δ [ppm] = 24.0 (d, ^3^*J*= 6.0 Hz, NCH(*C*H_3_)_2_), 24.7 (d, ^3^*J* = 8.0 Hz, NCH(*C*H_3_)_2_), 29.5 (d, ^3^*J* = 7.5 Hz, OC(*C*H_3_)_3_), 49.5 (dd, ^2^*J* = 10.5 Hz, *^3^J* = 6.0 Hz, N*C*H(CH_3_)_2_), 77.9 (s, O*C*(CH_3_)_3_).

**^31^P{^1^H} NMR (202 MHz, C_6_D_6_, 300 K):** δ [ppm] = 81.3 (dtd, ^1^*J*_AM_ = −184.9 Hz, ^2^*J*_AX_ = 109.8 Hz, ^3^*J*_AB_ = 18.2 Hz, 1P, P_A_), −17.9 (dtd, ^1^*J*_BX_ = −203.4 Hz, ^2^*J*_BM_ = 248.7 Hz, ^3^*J*_AB_ = 18.2 Hz, 1P, P_B_), −249.0 (ddt, ^1^*J*_AM_ = −184.9 Hz, ^1^*J*_MX_ = −173.0 Hz, ^2^*J*_BM_ = 248.7 Hz, 1P, P_M_), −346.3 (ddd, ^1^*J*_BX_ = −203.4 Hz, ^1^*J*_MX_ = −173.0 Hz, ^2^*J*_AX_ = 109.8 Hz, 2P, P_X_).

**Elemental Analysis:** calcd. (%) for C_16_H_37_N_2_OP_5_ (Mw = 428.35 g·mol^-1^): C 44.86, H 8.71, N 6.54; found C 45.92, H 9.45, N 6.58

Despite repeated attempts, satisfactory elemental analysis could not be obtained.

**ESI(+)-MS** (1,2-difluorobenzene) m/z (%): **429.1673 (100) for [M^+^]_._**

**Figure S21.** ^1^H NMR spectrum (500 MHz, C_6_D_6_, 300 K) of *exo,exo*-P_4_[P{N(*^i^*Pr)_2_}_2_](O*^t^*Bu) (**4b**); * = C_6_D_6_.

**Figure S22.** ^13^C{^1^H} NMR spectrum (126 MHz, C_6_D_6_, 300 K) of *exo,exo*-P_4_[P{N(*^i^*Pr)_2_}_2_](O*^t^*Bu) (**4b**); * = C_6_D_6_.

**Figure S23.** ^31^P{^1^H} NMR spectrum (202 MHz, C_6_D_6_, 300 K) of *exo,exo*-P_4_[P{N(*^i^*Pr)_2_}_2_](O*^t^*Bu) (**4b**).

**Table S4.** Coupling constants from the iterative fit of the ABMX_2_ spin system of *exo,exo*-P_4_[P{N(*^i^*Pr)_2_}_2_](O*^t^*Bu) (**4b**); δ (P_A_) = 81.3 ppm, δ (P_B_) = −17.9 ppm, δ (P_M_) = −249.0 ppm, δ (P_X_) = −346.3 ppm.

|  | ^1^*J*_BX_ = −203.4 Hz  ^1^*J*_AM_ = −184.9 Hz  ^1^*J*_MX_ = −173.0 Hz | ^2^*J*_BM_ = 248.7 Hz  ^2^*J*_AX_ = 109.8 Hz | ^3^*J*_AB_ = 18.2 Hz |
| --- | --- | --- | --- |

**Figure S24**. Sections of the ^31^P{^1^H} NMR spectrum (202.0 MHz, C_6_D_6_, 300 K) of *exo,exo*-P_4_[P{N(*^i^*Pr)_2_}_2_](O*^t^*Bu) (**4b**); experiment (upwards) and simulation (downwards).

**Figure S25.** ^31^P NMR spectrum (202 MHz, C_6_D_6_, 300 K) of *exo,exo*-P_4_[P{N(*^i^*Pr)_2_}_2_](O*^t^*Bu) (**4b**).

### Synthesis of P_4_[P{N(*^i^*Pr)_2_}_2_]{N(*^i^*Pr)_2_} (4c)

A solution of P_4_ (62 mg, 0.50 mmol, 1 equiv.) in THF (5 mL) was added to solid (6Dipp)CuN(*^i^*Pr)_2_ (285 mg, 0.50 mmol, 1 equiv.). The resulting orange solution was stirred for 15 minutes before the addition of bis(diisopropylamino)phosphorus chloride (133 mg, 0.50 mmol, 1 equiv.). The reaction mixture was stirred at room temperature for three days, during which a precipitate formed. ^31^P{^1^H} NMR spectroscopic analysis indicated the formation of two distinct isomers, *exo,exo*- P_4_[P{N(*^i^*Pr)_2_}_2_]{N(*^i^*Pr)_2_} and *endo,exo*- P_4_[P{N(*^i^*Pr)_2_}_2_]{N(*^i^*Pr)_2_}. The solvent was removed *in vacuo* and the products were extracted with *n*-pentane (3 × 3 mL). The combined orange extracts were filtered, concentrated and stored at −35 °C to yield orange crystals. The supernatant solution was decanted, the crystals were carefully washed with cold *n*-pentane and dried *in vacuo*. Recrystallisation from *n*-pentane afforded *exo,exo*-P_4_[P{N(*^i^*Pr)_2_}_2_]{N(*^i^*Pr)_2_} as a colourless crystalline solid.

**Yield:** 62 mg (0.14 mmol, 28%)

**^1^H NMR (500 MHz, C_6_D_6_, 300 K):** δ [ppm] = 0.94 (d, *J* = 6.8 Hz, 12H, NCH(C*H*_3_)_2_), 1.09 (d, *J* = 6.7 Hz, 12H, NCH(C*H*_3_)_2_), 1.30 (d, *J* = 6.7 Hz, 12H, NCH(C*H*_3_)_2_), 3.12 (sept, *J* = 6.8 Hz, 2H, NC*H*(CH_3_)_2_), 3.49 (sept, *J* = 6.7 Hz, 4H, NC*H*(CH_3_)_2_).

**^13^C{^1^H} NMR (126 MHz, C_6_D_6_, 300 K):** δ [ppm] = 23.2 (d, *J* = 7.0 Hz, NCH(*C*H_3_)_2_), 24.1 (d, *J* = 6.0 Hz, NCH(*C*H_3_)_2_), 24.8 (d, *J* = 7.5 Hz, NCH(*C*H_3_)_2_), 48.5 (d, *J* = 2.2 Hz, N*C*H(CH_3_)_2_), 49.6 (dd, *J* = 6.0 Hz, *J* = 10.0 Hz, N*C*H(CH_3_)_2_).

**^31^P{^1^H} NMR (202 MHz, C_6_D_6_, 300 K):** δ [ppm] = 81.7 (dtd, ^1^*J*_AM_ = −181.5 Hz, ^2^*J*_AX_ = 124.6 Hz, ^3^*J*_AB_ = 3.2 Hz, 1P, P_A_), −56.1 (dtd, ^1^*J*_BX_ = −191.4 Hz, ^2^*J*_BM_ = 252.1 Hz, ^3^*J*_AB_ = 3.2 Hz, 1P, P_B_), −217.9 (tdd, ^1^*J*_MX_ = −182.5 Hz, ^1^*J*_AM_ = −181.5 Hz, ^2^*J*_BM_ = 252.1 Hz, 1P, P_M_), −361.8 (ddd, ^1^*J*_BX_ = −191.4 Hz, ^1^*J*_MX_ = −182.5 Hz, ^2^*J*_AX_ = 124.6 Hz, 2P, P_X_).

**Elemental Analysis:** calcd. (%) for C_18_H_42_N_3_P_5_ (Mw = 455.42 g·mol^-1^): C 47.47, H 9.30, N 9.23; found C 48.81, H 10.04, N 9.04.

Despite repeated attempts, satisfactory elemental analysis could not be obtained.

**ESI(+)-MS (1,2-difluorobenzene) m/z (%):** **456.2141 (17) for [M^+^]**. Other observed fragments: 102.1277 (21) for [C_6_H_16_N]^+^, 249.2087 (7) for [C_12_H_30_N_2_OP]^+^, 350.3300 (100) for [C_18_H_45_N_3_OP]^+^, 497.4113 (45) for [C_24_H_59_N_4_O_2_P_2_]^+^. For proposed fragment formation see section III.

**Figure S26.** ^1^H NMR spectrum (500 MHz, C_6_D_6_, 300 K) of *exo,exo*-P_4_[P{N(*^i^*Pr)_2_}_2_]{N(*^i^*Pr)_2_} (**4c**); * = C_6_D_6_, ● = *n*-pentane.

**Figure S27.** ^13^C{^1^H} NMR spectrum (126 MHz, C_6_D_6_, 300 K) of *exo,exo*-P_4_[P{N(*^i^*Pr)_2_}_2_]{N(*^i^*Pr)_2_} (**4c**); * = C_6_D_6_, ● = *n*-pentane.

**Figure S28.** ^31^P{^1^H} NMR spectrum (202 MHz, C_6_D_6_, 300 K) of *exo,exo*-P_4_[P{N(*^i^*Pr)_2_}_2_]{N(*^i^*Pr)_2_} (**4c**).

**Table S5.** Coupling constants from the iterative fit of the ABMX_2_ spin system of *exo,exo*-P_4_[P{N(*^i^*Pr)_2_}_2_]{N(*^i^*Pr)_2_} (**4c**); δ (P_A_) = 81.7 ppm, δ (P_B_) = −56.1 ppm, δ (P_M_) = −217.9 ppm, δ (P_X_) = −361.8 ppm.

|  | ^1^*J*_BX_ = −191.4 Hz  ^1^*J*_MX_ = −182.5 Hz ^1^*J*_AM_ = −181.5 Hz | ^2^*J*_BM_ = 252.1 Hz  ^2^*J*_AX_ = 124.6 Hz | ^3^*J*_AB_ = 3.2 Hz |
| --- | --- | --- | --- |

**Figure S29.** Sections of the ^31^P{^1^H} NMR spectrum (202.0 MHz, C_6_D_6_, 300 K) of *exo,exo*-P_4_[P{N(*^i^*Pr)_2_}_2_]{N(*^i^*Pr)_2_} (**4c**); experiment (upwards) and simulation (downwards).

**Figure S30.** ^31^P NMR spectrum (202 MHz, C_6_D_6_, 300 K) of *exo,exo*-P_4_[P{N(*^i^*Pr)_2_}_2_]{N(*^i^*Pr)_2_} (**4c**).

**Figure S31.** ^31^P{^1^H} NMR spectrum (202 MHz, THF, 300 K) of the crude reaction mixture showing *exo,exo*-P_4_[P{N(*^i^*Pr)_2_}_2_]{N(*^i^*Pr)_2_} (blue) and *endo,exo*-P_4_[P{N(*^i^*Pr)_2_}_2_]{N(*^i^*Pr)_2_} (red); * = P_4_.

### Synthesis of *endo,exo*-P_4_(BPh_2_){N(*^i^*Pr)_2_} (5)

A light orange solution of [(6Dipp)Cu{κ^2^-P_4_N(*^i^*Pr)_2_}] (133 mg, 0.19 mmol, 1 equiv.) in toluene (4 mL) was added to a colourless solution of Ph_2_BCl (38.5 mg, 0.19 mmol, 1 equiv.) in toluene (1 mL). The deep orange solution was stirred for 1.5 h. The solvent was removed *in vacuo*, and the product was extracted in *n*-pentane (5 mL) and filtered. The filtrate was concentrated and stored at −35 °C overnight, resulting in light-yellow crystals. The supernatant solution was decanted, and the product was washed with cold *n*-pentane and dried *in vacuo*. Recrystallisation from *n*-pentane afforded *endo,exo*-P_4_(BPh_2_){N(*^i^*Pr)_2_} as colourless crystalline solid. The compound undergoes slow decomposition upon prolonged storage in solution.

**Yield:** 39.7 mg (0.10 mmol, 53%)

**^1^H NMR (500 MHz, C_6_D_6_, 300 K):** δ [ppm] = 0.66 (d, *J* = 6.8 Hz, 12H, CH(C*H*_3_)_2_), 2.79 (sept, *J* = 6.8 Hz, 1H, C*H*(CH_3_)_2_), 2.81 (sept, *J* = 6.8 Hz, 1H, C*H*(CH_3_)_2_), 7.20 – 7.23 (m, 2H, *p*-Ar–*H*), 7.30 –7.33 (m, 4H, *m*-Ar–*H*), 7.73 – 7.75 (m, 4H, *o*-Ar–*H*)

**^13^C{^1^H} NMR (126 MHz, C_6_D_6_, 300 K):** δ [ppm] = 23.2 (d, *J* = 2.9 Hz, NCH(*C*H_3_)_2_), 48.7 (s, N*C*H(CH_3_)_2_), 127.4 (d, *J* = 3.5 Hz, *p*-*C*–H, Ar), 128.0 (d, *J* = 1.9 Hz, *o*-*C*–H, Ar), 133.0 (dd, *J* = 6 Hz, *J* = 15 Hz, *m*-*C*–H, Ar), 149.4 (s, *i*-*C*, Ar)

**^31^P{^1^H} NMR (202 MHz, C_6_D_6_, 300 K):** δ [ppm] = −88.1 (td, ^1^*J*_AY_ = −254.4 Hz, ^2^*J*_AX_ = 56.3 Hz, 1P, P_A_), −260.9 (m,^1^*J*_XY_ = −193.1 Hz, ^2^*J*_AX_ = 56.3 Hz, 1P, P_X_), −279.6 (m, ^1^*J*_AY_ = −254.4 Hz, ^1^*J*_XY_ = −193.1 Hz, 2P, P_Y_).

**^11^B{^1^H} NMR (160 MHz, C_6_D_6_, 300 K):** δ [ppm] = 36.6 (br s)

**Elemental Analysis:** calcd. (%) for C_18_H_24_BNP_4_ (Mw = 389.10 g·mol^-1^): C 55.56, H 6.22, N 3.60; found C 56.94, H 6.55, N 3.58

Despite repeated attempts, satisfactory elemental analysis could not be obtained.

**FD-MS (*n*-hexane) m/z (%):** **389.0917 (100) for [M^+^]**.

**Figure S32.** ^1^H NMR spectrum (500 MHz, C_6_D_6_, 300 K) of *endo,exo*-P_4_(BPh_2_){N(*^i^*Pr)_2_} (**5**); * = C_6_D_6_.

**Figure S33.** ^13^C{^1^H} NMR spectrum (126 MHz, C_6_D_6_, 300 K) of *endo,exo*-P_4_(BPh_2_){N(*^i^*Pr)_2_} (**5**); * = C_6_D_6_.

**Figure S34.** ^31^P{^1^H} NMR spectrum (202 MHz, C_6_D_6_, 300 K) of *endo,exo*-P_4_(BPh_2_){N(*^i^*Pr)_2_} (**5**); * = P_4_.

**Table S6.** Coupling constants from the iterative fit of the AXY_2_ spin system of *endo,exo*-P_4_(BPh_2_){N(*^i^*Pr)_2_} (**5**); δ (P_A_) = −88.1 ppm, δ (P_X_) = −260.9 ppm, δ (P_Y_) = −279.6 ppm.

|  | ^1^*J*_AY_ = −254.4 Hz  ^1^*J*_XY_ = −193.1 Hz | ^2^*J*_AX_ = 56.3 Hz |
| --- | --- | --- |

**Figure S35.** Sections of the ^31^P{^1^H} NMR spectrum (202.0 MHz, C_6_D_6_, 300 K) of *endo,exo*-P_4_(BPh_2_){N(*^i^*Pr)_2_} (**5**); experiment (upwards) and simulation (downwards).

**Figure S36.** ^11^B NMR spectrum (160 MHz, C_6_D_6_, 300 K) of *endo,exo*-P_4_(BPh_2_){N(*^i^*Pr)_2_} (**5**).

### Synthesis of [*cyclo*-P_3_{N(*^i^*Pr)_2_}(P{N(*^i^*Pr)_2_}_2_)(P=IMes)] (6)

A solution of IMes (122 mg, 0.4 mmol, 2 equiv.) in toluene (3 mL) was added to a yellow solution of *exo,exo*-P_4_[P{N(*^i^*Pr)_2_}_2_]{N(*^i^*Pr)_2_} (91 mg, 0.2 mmol, 1 equiv.) in toluene (3 mL). The resulting bright green solution was stirred overnight, during which the colour changed to red-orange. The solvent was removed *in vacuo*, and the product was extracted in *n*-pentane (5 mL). Storage of the *n*-pentane extract at −35 °C gave red-orange crystals. The supernatant solution was decanted, the solid was washed with cold *n*-pentane (2 mL) and dried *in vacuo*.

**Yield:** 38 mg (0.05 mmol, 25%)

**^1^H NMR (500 MHz, C_6_D_6_, 300 K):** δ [ppm] = 1.08 (d, *J* = 6.7 Hz, 6H, CH(C*H*_3_)_2_), 1.13 (d, *J* = 6.7 Hz, 6H, CH(C*H*_3_)_2_), 1.18 (d, *J* = 6.7 Hz, 6H, CH(C*H*_3_)_2_), 1.25 (d, *J* = 6.7 Hz, 6H, CH(C*H*_3_)_2_), 1.33 (d, *J* = 6.7 Hz, 6H, CH(C*H*_3_)_2_), 1.40 (d, *J* = 6.7 Hz, 6H, CH(C*H*_3_)_2_), 2.15 (s, 6H, Ar-C*H*_3_), 2.20 (s, 6H, Ar-C*H*_3_), 2.29 (s, 6H, Ar-C*H*_3_), 3.09 (sept, *J* = 6.7 Hz, 2H, C*H*(CH_3_)_2_), 3.60 (sept, *J* = 6.7 Hz, 2H, C*H*(CH_3_)_2_), 4.13 (sept, *J* = 6.7 Hz, 2H, C*H*(CH_3_)_2_), 5.80 (s, 2H, N–C*H*), 6.71 (m, 2H, *m*-Ar–*H*), 6.81 (m, 2H, *m*-Ar–*H*)

**^13^C{^1^H} NMR (126 MHz, C_6_D_6_, 300 K):** δ [ppm] = 18.1 (Ar-*C*H_3_), 21.2 (Ar-*C*H*_3_*), 23.4 (CH(*C*H_3_)_2_), 23.8 (CH(*C*H_3_)_2_), 24.0 (CH(*C*H_3_)_2_), 24.4 (CH(*C*H_3_)_2_), 25.2(CH(*C*H_3_)_2_), 26.2 (CH(*C*H_3_)_2_), 47.3 (*C*H(CH_3_)_2_), 48.3 (*C*H(CH_3_)_2_), 50.3 (*C*H(CH_3_)_2_), 118.9 (N–*C*H), 129.6 (*m*-*C*–H*)*,134.9 (quart. *C*_6_H_3_), 136.0 (quart. *C*_6_H_3_), 138.7 (quart. *C*_6_H_3_).

**^31^P{^1^H} NMR (202 MHz, C_6_D_6_, 300 K):** δ [ppm] = 72.0 (dddd, ^1^*J*_AX_ = −227.7 Hz, ^2^*J*_AM_ = 216.3 Hz, ^2^*J*_AS_ = 16.4 Hz, ^3^*J*_AP_ = 142.7 Hz, 1P, P_A_), −56.0 (dddd, ^1^*J*_MS_ = −225.2 Hz, ^1^*J*_MX_ = −201.1 Hz, ^2^*J*_AM_ = 216.3 Hz, ^2^*J*_MP_ = 110.8 Hz, 1P, P_M_), −85.1 (dddd, ^1^*J*_PS_ = −291.7 Hz, ^2^*J*_MP_ = 110.8 Hz, ^2^*J*_PX_ = 6.1 Hz, ^3^*J*_AP_ = 142.7 Hz, 1P, P_P_), −141.7 (dddd, ^1^*J*_PS_ = −291.7 Hz, ^1^*J*_MS_ = −225.2 Hz, ^1^*J*_SX_ = −142.0 Hz, ^2^*J*_AS_ = 16.4 Hz, 1P, P_S_), −182.3 (dddd, ^1^*J*_AX_ = −227.7 Hz, ^1^*J*_MX_ = −201.1 Hz, ^1^*J*_SX_ = −142.0 Hz, ^2^*J*_PX_ = 6.1 Hz, 1P, P_X_)

Despite repeated attempts at purification, an analytically pure sample of **6** could not be obtained due to the similar solubilities of IMes, **4c** and the product. Using two equivalents of IMes was necessary to achieve satisfactory conversion; lower amounts resulted in incomplete consumption of **4c**, which was found to co-crystallise with the product. Washing with *n*-pentane proved to be the only viable method for partially removing residual IMes, but this resulted in a significant product loss. Alternative purification methods, including sublimation and column chromatography, were investigated but did not lead to satisfactory separation. The yield of **6** was determined by ^1^H NMR spectroscopy (see Figure S42).

**Figure S37.** ^1^H NMR spectrum (500 MHz, C_6_D_6_, 300 K) of **6** (pink) and 0.23 equiv. of IMes (blue);● = *n*-pentane, * = C_6_D_6_.

**Figure S38.** ^13^C{^1^H} NMR spectrum (126 MHz, C_6_D_6_, 300 K) of **6** (pink) and IMes (blue); ● = *n*-pentane,* = C_6_D_6_.

**Figure S39.** ^31^P{^1^H} NMR spectrum (202 MHz, C_6_D_6_, 300 K) of **6**.

**Table S7.** Coupling constants from the iterative fit of the AMPSX spin system of [*cyclo*-P_3_{N(*^i^*Pr)_2_}(P{N(*^i^*Pr)_2_}_2_)(P=IMes)] (**6**); δ (P_A_) = 72.0 ppm, δ (P_M_) = −56.0 ppm, δ (P_P_) = −85.1 ppm, δ (P_S_) = −141.7 ppm, δ (P_X_) = −182.3 ppm.

|  | ^1^*J*_PS_ = −291.7 Hz  ^1^*J*_AX_ = −227.7 Hz  ^1^*J*_MS_ = −225.2 Hz  ^1^*J*_MX_ = −201.1 Hz  ^1^*J*_SX_ = −142.0 Hz | ^2^*J*_AM_ = 216.3 Hz  ^2^*J*_MP_ = 110.8 Hz  ^2^*J*_AS_ = 16.4 Hz  ^2^*J*_PX_ = 6.1 Hz | ^3^*J*_AP_ = 142.7 Hz |
| --- | --- | --- | --- |

**Figure S40.** Sections of the ^31^P{^1^H} NMR spectrum (202.0 MHz, C_6_D_6_, 300 K) of **6**; experiment (upwards) and simulation (downwards).

**Figure S41.** ^31^P NMR spectrum (202 MHz, C_6_D_6_, 300 K) of **6**.

**Figure S42.** ^1^H NMR spectrum (500 MHz, C_6_D_6_, 300 K) of crude **6** (pink) after crystallisation containing 2.4 equiv. of IMes (blue); ● = *n*-pentane, * = C_6_D_6_.

**Figure S43.** ^31^P{^1^H} NMR spectrum (202 MHz, C_6_D_6_, 300 K) of crude **6** after crystallisation.

**Figure S44.** ^31^P{^1^H} NMR spectrum (202 MHz, C_6_D_6_, 300 K) of the reaction of **4c** (blue) with IMes after 3 h, 1 d and 13 d; Formation of **6** (pink) and an alternative diastereomer (orange); * = P_4_.

**Figure S45.** ^31^P{^1^H} NMR spectrum (202 MHz, C_6_D_6_, 300 K) of isolated **6** in solution over 49 d.

### Synthesis of [W(CO)_5_{κ^1^-P_4_(P{N(*^i^*Pr)_2_}_2_)(N{*^i^*Pr}_2_)}] (7)

A colourless solution of [W(CO)_6_] (35 mg, 0.1 mmol, 1. equiv.) in THF (1 mL) was irradiated with UV-light (365 nm, 10 W) for 30 min while stirring. The solution turned deep yellow and was added to a solution of *exo,exo*-P_4_[P{N(*^i^*Pr)_2_}_2_]{N(*^i^*Pr)_2_} (46 mg, 0.1 mmol, 1 equiv.) in THF. The solution was stirred overnight. The solvent was removed *in vacuo* and the product was extracted in *n*-pentane (5 mL). Concentrating the *n*-pentane extract and storing it at −35 °C gave colourless crystals. The supernatant solution was decanted, and the solid was dried *in vacuo* to give [W(CO)_5_{κ^1^-P_4_(P{N(*^i^*Pr)_2_}_2_)(N{*^i^*Pr}_2_)}] as an off-white powder.

**Yield:** 32 mg (0.04 mmol, 41%).

**^1^H NMR (500 MHz, C_6_D_6_, 300 K):** δ [ppm] = 1.00 (d, *J* = 6.7 Hz, 12H, CH(C*H*_3_)_2_), 1.05 (d, *J* = 6.7 Hz, 12H, CH(C*H*_3_)_2_), 1.25 (d, *J* = 6.7 Hz, 12H, CH(C*H*_3_)_2_), 3.15 (sept, *J* = 6.7 Hz, 2H, C*H*(CH_3_)_2_), 3.45 (sept, *J* = 6.7 Hz, 4H, C*H*(CH_3_)_2_)

**^13^C{^1^H} NMR (126 MHz, C_6_D_6_, 300 K):** δ [ppm] = 23.2 (s, NCH(*C*H_3_)_2_), 24.0 (d, *J* = 6.0 Hz, NCH(*C*H_3_)_2_), 24.7 (d, *J* = 7.7 Hz, NCH(*C*H_3_)_2_), 49.7 (m, *J* = 5 Hz, N*C*H(CH_3_)_2_), 50.9 (s, N*C*H(CH_3_)_2_), 197.9 (t, J = 8.0 Hz, *C*O), 198.4 (d, J = 38.7 Hz, *C*O).

**^31^P{^1^H} NMR (202 MHz, C_6_D_6_, 300 K):** δ [ppm] = 8.4 (dtd, ^1^*J*_AM_ = −175.8 Hz, ^2^*J*_AX_ = 117.3 Hz, ^3^*J*_AE_ = 34.7 Hz, 1P, P_A_), −26.5 (tdd,^1^*J*_EX_ = −224.4 Hz, ^2^*J*_EM_ = 162.3 Hz, ^3^*J*_AE_ = 34.7 Hz, 1P, P_E_), −174.4 (tdd, ^1^*J*_MX_ = −192.8 Hz, ^1^*J*_AM_ = −175.8 Hz, ^2^*J*_EM_ = 162.3 Hz, ^1^*J*_WP_ = 290.8 Hz, 1P, P_M_), −290.5 (ddd, ^1^*J*_EX_ = −224.4 Hz, ^1^*J*_MX_ = −192.8 Hz, ^2^*J*_AX_ = 117.3 Hz, 2P, P_X_).

**Elemental Analysis:** C_23_H_42_N_3_O_5_P_5_W (Mw = 779.31 g·mol^-1^); calcd. (%) for C_23_H_42_N_3_O_5_P_5_W ∙ (*n*-pentane)_0.2_: C 36.33, H 5.64, N 5.29; found C 36.42, H 5.66, N 5.25.

The ¹H NMR spectrum and the elemental analysis of the isolated sample of **7** indicate the presence of 0.2 equivalents of *n*-pentane.

**Figure S46.** ^1^H NMR spectrum (500 MHz, C_6_D_6_, 300 K) of [W(CO)_5_{κ^1^-P_4_(P{N(*^i^*Pr)_2_}_2_)(N{*^i^*Pr}_2_)}] (**7**); ● = *n*-pentane, * = C_6_D_6_.

**Figure S47.** ^13^C{^1^H} NMR spectrum (126 MHz, C_6_D_6_, 300 K) of [W(CO)_5_{κ^1^-P_4_(P{N(*^i^*Pr)_2_}_2_)(N{*^i^*Pr}_2_)}] (**7**); ● = *n*-pentane, * = C_6_D_6_.

**Figure S48.** ^31^P{^1^H} NMR spectrum (202 MHz, C_6_D_6_, 300 K) of [W(CO)_5_{κ^1^-P_4_(P{N(*^i^*Pr)_2_}_2_)(N{*^i^*Pr}_2_)}] (**7**); * = unidentified impurity.

**Table S8.** Coupling constants from the iterative fit of the ABMX_2_ spin system of [W(CO)_5_{κ^1^-P_4_(P{N(*^i^*Pr)_2_}_2_)(N{*^i^*Pr}_2_)}] (**7**); δ (P_A_) = 8.4 ppm, δ (P_E_) = −26.5 ppm, δ (P_M_) = −174.4 ppm, δ (P_X_) = −290.5 ppm.

|  | ^1^*J*_WP_ = 290.8 Hz ^1^*J*_EX_ = −224.4 Hz  ^1^*J*_MX_ = −192.8 Hz  ^1^*J*_AM_ = −175.8 Hz | ^2^*J*_EM_ = 162.3 Hz ^2^*J*_AX_ = 117.3 Hz | ^3^*J*_AE_ = 34.7 Hz |
| --- | --- | --- | --- |

**Figure S49.** Sections of the ^31^P{^1^H} NMR spectrum (202.0 MHz, C_6_D_6_, 300 K) of [W(CO)_5_{κ^1^-P_4_(P{N(*^i^*Pr)_2_}_2_)(N{*^i^*Pr}_2_)}] (**7**); experiment (upwards) and simulation (downwards).

 **Figure S50.** ^31^P NMR spectrum (202 MHz, C_6_D_6_, 300 K) of [W(CO)_5_{κ^1^-P_4_(P{N(*^i^*Pr)_2_}_2_)(N{*^i^*Pr}_2_)}] (**7**).

### Synthesis of [CuBr{κ^2^-P_4_(P{N(*^i^*Pr)_2_}_2_)(N{*^i^*Pr}_2_)}] (8)

A white suspension of CuBr∙SMe_2_ (49 mg, 0.24 mmol, 1.2 equiv.) in toluene (3 mL) was added to a yellow solution of *exo,exo*-P_4_[P{N(*^i^*Pr)_2_}_2_]{N(*^i^*Pr)_2_} (91 mg, 0.2 mmol, 1 equiv.) in toluene (2 mL). The resulting dark brown solution was stirred at room temperature for 48 hours, after which the solvent was removed *in vacuo* and the product was extracted in *n*-pentane (5 mL) before being filtered. Concentrating the extract and storing it at −35 °C gave light-brown crystals. The supernatant solution was decanted, the solid was washed with cold *n*-pentane (2 mL) and dried *in vacuo*, yielding [CuBr{κ^2^-P_4_(P{N(*^i^*Pr)_2_}_2_)(N{*^i^*Pr}_2_)}] as a light brown powder.

**Yield:** 8.3 mg (0.02 mmol, 7%)

**^1^H NMR (500 MHz, C_6_D_6_, 300 K):** δ [ppm] = 1.16 (d, *J* = 6.8 Hz, 12H, CH(C*H*_3_)_2_), 1.20 (d, *J* = 6.8 Hz, 12H, CH(C*H*_3_)_2_), 1.24 (d, *J* = 6.8 Hz, 12H, CH(C*H*_3_)_2_), 3.41 (sept, *J* = 6.8 Hz, 6H, C*H*(CH_3_)_2_)

**^13^C{^1^H} NMR (126 MHz, C_6_D_6_, 300 K):** δ [ppm] = 23.1 (d, *J* = 4.0 Hz, NCH(*C*H_3_)_2_), 23.2 (d, *J* = 4.4 Hz, NCH(*C*H_3_)_2_), 25.3 (d, *J* = 3.7 Hz, NCH(*C*H_3_)_2_), 49.9 (m, N*C*H(CH_3_)_2_), 50.6 (m, N*C*H(CH_3_)_2_).

**^31^P{^1^H} NMR (202 MHz, C_6_D_6_, 300 K):** δ [ppm] = 30.6 (dd, ^1^*J*_AM_ = −189.8 Hz, ^2^*J*_AB_ = 454.8 Hz, 1P, P_A_), 7.5 (dtd,^1^*J*_BX_ = −248.6 Hz, ^2^*J*_AB_ = 454.8 Hz, ^2^*J*_BM_ = 18.6 Hz, 1P, P_B_), −156.0 (tdd, ^1^*J*_MX_ = −278.8 Hz, ^1^*J*_AM_ = −189.8 Hz, ^2^*J*_BM_ = 18.6 Hz, 1P, P_M_) −303.1 (dd, ^1^*J*_MX_ = −278.8 Hz, ^1^*J*_BX_ = −248.6 Hz, 2P, P_X_).

The reaction did not proceed to full conversion under any of the examined conditions. Prolonged reaction times increased product formation but were accompanied by gradual decomposition. Heating the reaction mixture to 50 °C resulted in rapid decomposition. Using increased amounts of CuBr∙SMe_2_ (1.5 or 2.0 equiv.) improved conversion, but substantial product decomposition was observed under these conditions.

**Figure S51.** ^1^H NMR spectrum (500 MHz, C_6_D_6_, 300 K) of [CuBr{κ^2^-P_4_(P{N(*^i^*Pr)_2_}_2_)(N{*^i^*Pr}_2_)}] (**8**); * = C_6_D_6_.

**Figure S52.** ^13^C{^1^H} NMR spectrum (126 MHz, C_6_D_6_, 300 K) of [CuBr{κ^2^-P_4_(P{N(*^i^*Pr)_2_}_2_)(N{*^i^*Pr}_2_)}] (**8**); * = C_6_D_6_.

**Figure S53.** ^31^P{^1^H} NMR spectrum (202 MHz, C_6_D_6_, 300 K) of [CuBr{κ^2^-P_4_(P{N(*^i^*Pr)_2_}_2_)(N{*^i^*Pr}_2_)}] (**8**); * = P_4_.

**Table S9.** Coupling constants from the iterative fit of the ABMX_2_ spin system of [CuBr{κ^2^-P_4_(P{N(*^i^*Pr)_2_}_2_)(N{*^i^*Pr}_2_)}] (**8**); δ (P_A_) = 30.6 ppm, δ (P_B_) = 7.5 ppm, δ (P_M_) = −156.0 ppm, δ (P_X_) = −303.1 ppm.

|  | ^1^*J*_MX_ = −278.8 Hz ^1^*J*_BX_ = −248.6 Hz ^1^*J*_AM_ = −189.8 Hz | ^2^*J*_AB_ = 454.8 Hz  ^2^*J*_BM_ = 18.6 Hz |
| --- | --- | --- |

**Figure S54.** Sections of the ^31^P{^1^H} NMR spectrum (202.0 MHz, C_6_D_6_, 300 K) of [CuBr{κ^2^-P_4_(P{N(*^i^*Pr)_2_}_2_)(N{*^i^*Pr}_2_)}] (**8**); experiment (upwards) and simulation (downwards).

## 4 NMR Scale Reactions

### Reaction of [(6Dipp)Cu(κ^2^-P_4_O*^t^*Bu)] with MeI

To a solution of [(6Dipp)Cu(κ^2^-P_4_O*^t^*Bu)] (33.3 mg, 0.05 mmol, 1 equiv.) in THF (0.7 mL) was added iodomethane (15.6 µL, 0.25 mmol, 5 equiv.) which caused immediate formation of a white precipitate. ^31^P{^1^H} NMR spectroscopic analysis of the crude reaction mixture indicated the exclusive formation of the *exo,exo*-P_4_(Me)(O*^t^*Bu) butterfly isomer. The product decomposed within 5 h in solution.

**^31^P{^1^H} NMR (202 MHz, THF, 300 K):** δ [ppm] = −21.1 (dt, 1P, ^1^*J*_AX_ = −214.4 Hz, ^2^*J*_AM_ = 284.7 Hz, P_A_), −231.0 (dt, 1P, ^1^*J*_MX_ = −177.5 Hz, ^2^*J*_AM_ = 284.7 Hz, P_M_), −339.5 (dd, 2P, ^1^*J*_AX_ = −214.4 Hz, ^1^*J*_MX_ = −177.5 Hz, P_X_).

**Figure S55.** ^31^P{^1^H} NMR spectrum (202 MHz, THF, 300 K) of the reaction of [(6Dipp)Cu(κ^2^-P_4_O*^t^*Bu)] with MeI; formation of *exo,exo*-P_4_(Me)(O*^t^*Bu) (blue); * = P_4_.

**Table S10.** Coupling constants from the iterative fit of the AXY_2_ spin system of *exo,exo*-P_4_(Me)(O*^t^*Bu); δ (P_A_) = −21.1 ppm, δ (P_X_) = −231.0 ppm, δ (P_Y_) = −339.5 ppm.

|  | ^1^*J*_AX_ = −214.4 Hz  ^1^*J*_MX_ = −177.5 Hz | ^2^*J*_AM_ = 284.7 Hz |
| --- | --- | --- |

**Figure S56.** Sections of the ^31^P{^1^H} NMR spectrum (202 MHz, THF, 300 K) of *exo,exo*-P_4_(Me)(O*^t^*Bu); experiment (upwards) and simulation (downwards).

### Reaction of [(6Dipp)Cu(κ^2^-P_4_O*^t^*Bu)] with Ad(CO)Cl

To a solution of [(6Dipp)Cu(κ^2^-P_4_O*^t^*Bu)] (33.3 mg, 0.05 mmol, 1 equiv.) in THF (0.7 mL) was added adamantanecarbonyl chloride (9.9 mg, 0.05 mmol, 1 equiv.), resulting in a pale yellow solution. ^31^P{^1^H} NMR spectroscopic analysis of the crude reaction mixture indicated the formation of two butterfly isomers: *exo,exo*-P_4_(COAd)(O*^t^*Bu) as the major product (94%) and *endo,exo*-P_4_(COAd)(O*^t^*Bu) as the minor product (6%). The product mixture completely decomposed within five days in solution.

***exo,exo*: ^31^P{^1^H} NMR (202 MHz, THF, 300 K):** δ [ppm] = −20.1 (dt, ^1^*J*_AX_ = −198.9 Hz, ^2^*J*_AM_ = 257.8 Hz, 1P, P_A_), −215.1 (dt, ^1^*J*_MX_ = −172.4 Hz, ^2^*J*_AM_ = 257.8 Hz, 1P, P_M_), −330.6 (dd, ^1^*J*_AX_ = −198.9 Hz, ^1^*J*_MX_ = −172.4 Hz, 2P, P_X_).

***endo,exo*: ^31^P{^1^H} NMR (202 MHz, THF, 300 K):** δ [ppm] = −12.1 (dt, ^1^*J*_AX_ = −228.2 Hz, ^2^*J*_AM_ = 13.6 Hz, 1P, P_A_), −192.0 (dt, ^1^*J*_MX_ = −264.5 Hz, ^2^*J*_AM_ = 13.6 Hz, 1P, P_M_), −303.6 (dd, ^1^*J*_MX_ = −264.5 Hz, ^1^*J*_AX_ = −228.2 Hz, 2P, P_X_).

**Figure S57.** ^31^P{^1^H} NMR spectrum (202 MHz, THF, 300 K) of the reaction of [(6Dipp)Cu(κ^2^-P_4_O*^t^*Bu)] with Ad(CO)Cl, formation of *exo,exo*-P_4_(COAd)(O*^t^*Bu) (blue) and *endo,exo*-P_4_(COAd)(O*^t^*Bu) (red); * = P_4_.

**Table S11.** Coupling constants from the iterative fit of the AMX_2_ spin system of *exo,exo*-P_4_(COAd)(O*^t^*Bu); δ (P_A_) = −20.1 ppm, δ (P_M_) = −215.1 ppm, δ (P_X_) = −330.6 ppm.

|  | ^1^*J*_AX_ = −198.9 Hz  ^1^*J*_MX_ = −172.4 Hz | ^2^*J*_AM_ = 257.8 Hz |
| --- | --- | --- |

**Figure S58.** Sections of the ^31^P{^1^H} NMR spectrum (202.0 MHz, THF, 300 K) of *exo,exo*-P_4_(COAd)(O*^t^*Bu); experiment (upwards) and simulation (downwards).

**Table S12.** Coupling constants from the iterative fit of the AMX_2_ spin system of *endo,exo*-P_4_(COAd)(O*^t^*Bu); δ (P_A_) = −12.1 ppm, δ (P_M_) = −192.0 ppm, δ (P_X_) = −303.6 ppm.

|  | ^1^*J*_MX_ = −264.5 Hz  ^1^*J*_AX_ = −228.2 Hz | ^2^*J*_AM_ = 13.6 Hz |
| --- | --- | --- |

**Figure S59.** Sections of the ^31^P{^1^H} NMR spectrum (202.0 MHz, THF, 300 K) of *endo,exo*-P_4_(COAd)(O*^t^*Bu); experiment (upwards) and simulation (downwards).

### Reaction of [(6Dipp)Cu(κ^2^-P_4_O*^t^*Bu)] with *^t^*Bu(CO)Cl

To a solution of [(6Dipp)Cu(κ^2^-P_4_O*^t^*Bu)] (33.3 mg, 0.05 mmol, 1 equiv.) in THF (0.7 mL) was added pivaloyl chloride (6.1 µL, 0.05 mmol, 1 equiv.), resulting in a pale yellow solution. ^31^P{^1^H} NMR analysis indicated the formation of two butterfly compounds: *exo,exo*-P_4_(CO*^t^*Bu)(O*^t^*Bu) as the major product (95%) and *endo,exo*-P_4_(CO*^t^*Bu)(O*^t^*Bu) as the minor product (5%). The product mixture was found to decompose into unidentified phosphorus-containing species after one day in solution.

***exo,exo*: ^31^P{^1^H} NMR (162 MHz, THF, 300 K):** δ [ppm] = −18.4 (dt, ^1^*J*_AX_ = −199.1 Hz, ^2^*J*_AM_ = 257.1 Hz, 1P, P_A_), −211.5 (dt, ^1^*J*_MX_ = −175.6 Hz, ^2^*J*_AM_ = 257.1 Hz, 1P, P_M_), −324.6 (dd, ^1^*J*_AX_ = −199.1 Hz, ^1^*J*_MX_ = −175.6 Hz, 2P, P_X_).

***endo,exo*: ^31^P{^1^H} NMR (162 MHz, THF, 300 K):** δ [ppm] = −9.5 (td, ^1^*J*_AX_ = −228.2 Hz, ^2^*J*_AM_ = 13.1 Hz, 1P, P_A_), −187.2 (td, ^1^*J*_MX_ = −266.3 Hz, ^2^*J*_AM_ = 13.1 Hz, 1P, P_M_), −299.5 (dd, ^1^*J*_AX_ = −228.2 Hz, ^1^*J*_MX_ = −266.3 Hz, 2P, P_X_).

**Figure S60.** ^31^P{^1^H} NMR spectrum (202 MHz, THF, 300 K) of the reaction of [(6Dipp)Cu(κ^2^-P_4_O*^t^*Bu)] with *^t^*Bu(CO)Cl**,** formation of *exo,exo*-P_4_(CO*^t^*Bu)(O*^t^*Bu) (blue) and *endo,exo*-P_4_(CO*^t^*Bu)(O*^t^*Bu) (red); * = P_4_.

**Table S13.** Coupling constants from the iterative fit of the AMX_2_ spin system of *exo,exo*-P_4_(CO*^t^*Bu)(O*^t^*Bu); δ (P_A_) = −18.4 ppm, δ (P_M_) = −211.5 ppm, δ (P_X_) = −324.6 ppm.

|  | ^1^*J*_AX_ = −199.1 Hz  ^1^*J*_MX_ = −175.6 Hz | ^2^*J*_AM_ = 257.1 Hz |
| --- | --- | --- |

**Figure S61.** Sections of the ^31^P{^1^H} NMR spectrum (202.0 MHz, THF, 300 K) of *exo,exo*-P_4_(CO*^t^*Bu)(O*^t^*Bu); experiment (upwards) and simulation (downwards).

**Table S14.** Coupling constants from the iterative fit of the AMX_2_ spin system of *endo,exo*-P_4_(CO*^t^*Bu)(O*^t^*Bu); δ (P_A_) = −9.5 ppm, δ (P_M_) = −187.2 ppm, δ (P_X_) = −299.5 ppm.

|  | ^1^*J*_MX_ = −266.3 Hz  ^1^*J*_AX_ = −228.2 Hz | ^2^*J*_AM_ = 13.1 Hz |
| --- | --- | --- |

**Figure S62**. Sections of the ^31^P{^1^H} NMR spectrum (202.0 MHz, THF, 300 K) of *endo,exo*-P_4_(CO*^t^*Bu)(O*^t^*Bu); experiment (upwards) and simulation (downwards).

### Reaction of [(6Dipp)Cu(κ^2^-P_4_O*^t^*Bu)] with Cy(CO)Cl

To a solution of [(6Dipp)Cu(κ^2^-P_4_O*^t^*Bu)] (33.3 mg, 0.05 mmol, 1 equiv.) in THF (0.7 mL) was added cyclohexanecarbonyl chloride (6.6 µL, 0.05 mmol, 1 equiv.), resulting in a pale yellow solution. ^31^P{^1^H} NMR analysis indicated the formation of two butterfly compounds: *exo,exo*-P_4_(COCy)(O*^t^*Bu) as the major product (86%) and *endo,exo*-P_4_(COCy)(O*^t^*Bu) as the minor product (14%). The product mixture was found to decompose into unidentified phosphorus-containing species after one day in solution.

***exo,exo*: ^31^P{^1^H} NMR (162 MHz, THF, 300 K):** δ [ppm] = −19.8 (dt, ^1^*J*_AX_ = −201.2 Hz, ^2^*J*_AM_ = 249.7 Hz, 1P, P_A_), −215.5 (dt,^1^*J*_MX_ = −172.3 Hz, ^2^*J*_AM_ = 249.7 Hz, 1P, P_M_), −330.6 (dd, ^1^*J*_AX_ = −201.2 Hz, ^1^*J*_MX_ = −172.3 Hz, 2P, P_X_).

***endo,exo*: ^31^P{^1^H} NMR (162 MHz, THF, 300 K):** δ [ppm] = −12.9 (td, ^1^*J*_AX_ = −227.1 Hz, ^2^*J*_AM_ = 12.6 Hz, 1P, P_A_), −190.4 (td,^1^*J*_MX_ = −262.9 Hz, ^2^*J*_AM_ = 12.6 Hz, 1P1, P_M_), −301.3 (dd, ^1^*J*_AX_ = −227.1 Hz, ^1^*J*_MX_ = −262.9 Hz, 2P, P_X_).

**Figure S63.** ^31^P{^1^H} NMR spectrum (162 MHz, THF, 300 K) of *exo,exo*-P_4_(COCy)(O*^t^*Bu) (blue) and *endo,exo*-P_4_(COCy)(O*^t^*Bu) (red); * = P_4_.

**Table S15.** Coupling constants from the iterative fit of the AMX_2_ spin system of *exo,exo*-P_4_(COCy)(O*^t^*Bu); δ (P_A_) = −19.8 ppm, δ (P_M_) = −215.5 ppm, δ (P_X_) = −330.6 ppm.

|  | ^1^*J*_AX_ = −201.2 Hz  ^1^*J*_MX_ = −172.3 Hz | ^2^*J*_AM_ = 249.7 Hz |
| --- | --- | --- |

**Figure S64.** Sections of the ^31^P{^1^H} NMR spectrum (202.0 MHz, THF, 300 K) of *exo,exo*-P_4_(COCy)(O*^t^*Bu); experiment (upwards) and simulation (downwards).

**Table S16.** Coupling constants from the iterative fit of the AMX_2_ spin system of *endo,exo*-P_4_(COCy)(O*^t^*Bu); δ (P_A_) = −12.9 ppm, δ (P_M_) = −190.4 ppm, δ (P_X_) = −301.3 ppm.

|  | ^1^*J*_AX_ = −227.1 Hz  ^1^*J*_MX_ = −262.9 Hz | ^2^*J*_AM_ = 12.6 Hz |
| --- | --- | --- |

**Figure S65.** Sections of the ^31^P{^1^H} NMR spectrum (202.0 MHz, THF, 300 K) of *endo,exo*-P_4_(COCy)(O*^t^*Bu); experiment (upwards) and simulation (downwards).

### Synthesis of *exo,exo*-P_4_(PPh_2_)(O*^t^*Bu)

To a solution of [(6Dipp)Cu(κ^2^-P_4_O*^t^*Bu)] (33.3 mg, 0.05 mmol, 1 equiv.) in THF (0.7 mL) was added diphenylphosphorus chloride (9.2 µL, 0.05 mmol, 1 equiv.) which led to the immediate formation of a white precipitate. ^31^P{^1^H} NMR spectroscopic analysis indicated the exclusive formation of the *exo,exo*-P_4_(PPh_2_)(O*^t^*Bu) butterfly isomer. The product mixture was found to decompose overnight in solution.

**^31^P{^1^H} NMR (162 MHz, THF, 300 K):** δ [ppm] = 2.7 (dtd, ^1^*J*_AX_ = −199.1 Hz, ^2^*J*_AM_ = 282.0 Hz, ^3^*J*_AB_ = 12.8 Hz, 1P, P_A_), −4.8 (dtd, ^1^*J*_BM_ = −182.8 Hz, ^2^*J*_BX_ = 88.1 Hz, ^3^*J*_AB_ = 12.8 Hz, 1P, P_B_), −238.9 (ddt, ^1^*J*_BM_ = −182.8 Hz, ^1^*J*_MX_ = −166.3 Hz, ^2^*J*_AM_ = 282.0 Hz, 1P, P_M_), −338.3 (ddd, ^1^*J*_AX_ = −199.1 Hz, ^1^*J*_MX_ = −166.3 Hz, ^2^*J*_BX_ = 88.1 Hz, 2P, P_X_).

**Figure S66.** ^31^P{^1^H} NMR spectrum (162 MHz, THF, 300 K) of *exo,exo*-P_4_(PPh_2_)(O*^t^*Bu) (blue); ■ = Ph_2_PCl, ○ = (PPh_2_)_2_, * = P_4_.

**Table S17.** Coupling constants from the iterative fit of the ABMX_2_ spin system of *exo,exo*- P_4_(PCy_2_)(O*^t^*Bu); δ (P_A_) = 2.7 ppm, δ (P_B_) = −4.8 ppm, δ (P_M_) = −238.9 ppm, δ (P_X_) = −338.3 ppm.

|  | ^1^*J*_AX_ = −199.1 Hz  ^1^*J*_BM_ = −182.8 Hz  ^1^*J*_MX_ = −166.3 Hz | ^2^*J*_AM_ = 282.0 Hz  ^2^*J*_BX_ = 88.1 Hz | ^3^*J*_AB_ = 12.8 Hz |
| --- | --- | --- | --- |

**Figure S67.** Sections of the ^31^P{^1^H} NMR spectrum (202.0 MHz, THF, 300 K) of *exo,exo*-P_4_(PPh_2_)(O*^t^*Bu); experiment (upwards) and simulation (downwards).

### Synthesis of *exo,exo*-P_4_(PCy_2_)(O*^t^*Bu)

To a solution of [(6Dipp)Cu(κ^2^-P_4_O*^t^*Bu)] (33.3 mg, 0.05 mmol, 1 equiv.) in THF (0.7 mL) was added dicyclohexylphosphorus chloride (11.0 µL, 0.05 mmol, 1 equiv.) which led to the immediate formation of a white precipitate. ^31^P{^1^H} NMR analysis indicated the exclusive formation of the *exo,exo*-P_4_(PCy_2_)(O*^t^*Bu) butterfly isomer. The product mixture was found to decompose after 2 days in solution.

**^31^P{^1^H} NMR (162 MHz, THF, 300 K):** δ [ppm] = 20.9 (dtd, ^1^*J*_AM_ = −179.6 Hz, ^2^*J*_AX_ = 81.3 Hz, ^3^*J*_AB_ = 17.9 Hz, 1P, P_A_), 1.8 (dtd, ^1^*J*_BX_ = −196.6 Hz, ^2^*J*_BM_ = 282.6 Hz, ^3^*J*_AB_ = 17.9 Hz, 1P, P_B_), −261.0 (ddt, ^1^*J*_AM_ = −179.6 Hz, ^1^*J*_MX_ = −164.6 Hz, ^2^*J*_BM_ = 282.6 Hz, 1P, P_M_), −343.4 (ddd, ^1^*J*_BX_ = −196.6 Hz, ^1^*J*_MX_ = −164.6 Hz, ^2^*J*_AX_ = 81.3 Hz, 2P, P_X_).

**Figure S68.** ^31^P{^1^H} NMR spectrum (202 MHz, THF, 300 K) of *exo,exo*-P_4_(PCy_2_)(O*^t^*Bu) (blue); ● = Cy_2_PCl, * = P_4_.

**Table S18.** Coupling constants from the iterative fit of the ABMX_2_ spin system of *exo,exo*- P_4_(PCy_2_)(O*^t^*Bu); δ (P_A_) = 20.9 ppm, δ (P_B_) = 1.8 ppm, δ (P_M_) = −261.0 ppm, δ (P_X_) = −343.4 ppm.

|  | ^1^*J*_BX_ = −196.6 Hz  ^1^*J*_AM_ = −179.6 Hz ^1^*J*_MX_ = −164.6 Hz | ^2^*J*_BM_ = 282.6 Hz  ^2^*J*_AX_ = 81.3 Hz | ^3^*J*_AB_ = 17.9 Hz |
| --- | --- | --- | --- |

**Figure S69.** Sections of the ^31^P{^1^H} NMR spectrum (202.0 MHz, THF, 300 K) of *exo,exo*-P_4_(PCy_2_)(O*^t^*Bu); experiment (upwards) and simulation (downwards).

## 5 Additional Experiments

### Recovery of (6Dipp)CuCl

To a solution of [(6Dipp)Cu(κ^2^-P_4_O*^t^*Bu)] (50 mg, 0.075 mmol, 1 equiv.) in THF (4 mL) was added a colourless solution of bis(2,4,6-trimethylphenyl)phosphorus chloride (27.4 mg, 0.09 mmol, 1.2 equiv.) in THF (1 mL). After 1 hour of stirring, the volatiles were removed *in vacuo*. The solid was washed with *n*-hexane (2 × 2 mL) and dried *in vacuo*. (6Dipp)CuCl was isolated as a beige solid.

**Yield:** 29 mg (0.06 mmol, 77%)

**^1^H NMR (400 MHz, C_6_D_6_, 300 K):** δ [ppm] = 1.16 (d, *J* = 6.9 Hz, 12H, CH(C*H*_3_)_2_), 1.44 (p, *J* = 5.9 Hz, 2H, CH_2_–C*H*_2_–CH_2_), 1.46 (d, *J* = 6.9 Hz, 12H, CH(C*H*_3_)_2_), 2.68 (t, *J* = 5.9 Hz, 4H, N–C*H*_2_), 2.99 (sept, *J* = 6.9 Hz, 4H, C*H*(CH_3_)_2_), 7.04 – 7.06 (m, 4H, *m*-Ar–*H*), 7.16 – 7.19 (m, 2H, *p*-Ar–*H*).

**Figure S70.** ^1^H NMR spectrum (400 MHz, C_6_D_6_, 300 K) of the recovered (6Dipp)CuCl; * = C_6_D_6_.

### Synthesis of [(IPr)Cu(κ^2^-P_4_O*^t^*Bu)]

A solid mixture of (IPr)CuO*^t^*Bu (26.3 mg, 0.05 mmol, 1 equiv.) and P_4_ (6.2 mg, 0.05 mmol, 1 equiv.) was dissolved in C_6_D_6_ (0.5 mL). The resulting bright orange reaction mixture was then transferred to a J. Young’s NMR tube and analysed by multinuclear NMR spectroscopy. ^1^H NMR and ^31^P{^1^H} NMR spectroscopic analysis indicated the formation of [(IPr)Cu(κ^2^-P_4_O*^t^*Bu)]. The product was found to slowly decompose within 3 days in solution.

**^1^H NMR (400 MHz, C_6_D_6_, 300 K):** δ [ppm] = 1.07 (s, 9H, O(C*H*_3_)_3_), 1.08 (d, *J* = 6.8 Hz, 12H, CH(C*H*_3_)_2_), 1.47 (d, *J* = 6.8 Hz, 12H, CH(C*H*_3_)_2_), 2.64 (sept, *J* = 6.8 Hz, 4H, C*H*(CH_3_)_2_), 6.31 (s, 2H, NC*H*), 7.11 – 7.13 (m, 4H, *m*-Ar–*H*), 7.22 – 7.26 (m, 2H, *p*-Ar–*H*).

**^31^P{^1^H} NMR (162 MHz, C_6_D_6_, 300 K):** δ [ppm] = −159.8 (q, ^1^*J*_PP_ = −131 Hz, 1P, P_A_), −347.9 (d, ^1^*J*_PP_ = −131 Hz, 3P, P_X_/P_Y_).

**Figure S71.** ^1^H NMR spectrum (400 MHz, C_6_D_6_, 300 K) of [(IPr)Cu(κ^2^-P_4_O*^t^*Bu)]; * = C_6_D_6_.

**Figure S72.** ^31^P{^1^H} NMR spectrum (162 MHz, C_6_D_6_, 300 K) of [(IPr)Cu(κ^2^-P_4_O*^t^*Bu)]; * = P_4_.

**Figure S73.** ^1^H NMR spectrum (400 MHz, C_6_D_6_, 300 K) of [(IPr)Cu(κ^2^-P_4_O*^t^*Bu)] measured immediately after the reaction start (top) and after 3 days (bottom); * = C_6_D_6_.

**Figure S74.** ^31^P{^1^H} NMR spectrum (162 MHz, C_6_D_6_, 300 K) of [(IPr)Cu(κ^2^-P_4_O*^t^*Bu)] measured immediately after the reaction start (top) and after 3 days (bottom); * = P_4_.

### Reaction of *exo,exo*-P_4_[P{N(*^i^*Pr)_2_}_2_]{N(*^i^*Pr)_2_} (4c) with TMC (1,3,4,5-tetramethyl-imidazolin-2-ylidene)

A solution of **4c** (11.4 mg, 0.025 mmol, 1 equiv.) in THF (0.7 mL) was added to TMC (3.1 mg, 0.025 mmol, 1 equiv.) which caused immediate colour change to dark orange. ^31^P{^1^H} NMR spectroscopic analysis of the crude reaction mixture indicated the formation of a P_2_ NHC-phosphinidene adduct (TMC)P–P{N(*i*Pr)_2_}_2_, the previously reported P_1_ cation [(TMC)_2_P]^+^,^[7]^ and other unidentified side products. The reaction mixture was dried and extracted into hexane, from which single crystals of (TMC)P–P{N(*i*Pr)_2_}_2_ were grown, allowing characterisation by XRD (see Figure S97).

**(TMC)P–P{N(*i*Pr)_2_}_2_: ^31^P{^1^H} NMR (202 MHz, THF, 300 K):** δ [ppm] = 74.1 (d, ^1^*J*_PP_ = −293 Hz, 1P, P_A_), −100.6 (d, ^1^*J*_PP_ = −293 Hz, 1P, P_M_).

**[(TMC)_2_P]^+^:** **^31^P{^1^H} NMR (202 MHz, THF, 300 K):** δ [ppm] = −113.0 (s, 1P).

**Figure S75.** ^31^P{^1^H} NMR spectrum (202 MHz, THF, 300 K) of the reaction of **4c** with TMC; * = (TMC)P–P{N(*i*Pr)_2_}_2_, ○ = [(TMC)_2_P]^+^, ● = unidentified product.

# II. Line-shape Analysis

Rate constants at each temperature were calculated using the full line shape iteration feature of gNMR.^[5]^ The static line width was evaluated at slow exchange (198 K). The chemical shifts and coupling constants were freely refined at that temperature. For all other temperatures, the coupling constants and static line widths were fixed.

The relationship between the rate constant *k* and the Gibbs energy of activation ${\Delta G}^{\ddagger}$is established by the Eyring equation **(1)**:

$k=\kappa\frac{k_{B}T}{h}\exp(-\frac{{\Delta G}^{\ddagger}}{RT})$	**(1)**

where *k_B_* is the Boltzmann constant, *T* is the absolute temperature, *h* is the Planck constant, $\kappa$ is the transmission constant and *R* is the gas constant.

By applying the Gibbs-Helmholtz equation **(2)** and linearising equation **(1)**, the parameters ${\Delta G}^{\ddagger}$ and $\kappa$ can be determined by a least-squares fitting procedure:

Δ𝐺 = Δ𝐻 – 𝑇Δ𝑆	**(2)**

$ln\left( \frac{k}{T} \right) = - \frac{{\Delta H}^{\ddagger}}{R}\cdot\frac{1}{T}+\frac{{\Delta S}^{\ddagger}}{R}+ln\left( \kappa\frac{k_{B}}{h} \right)$	**(3)**

The Eyring plot can be obtained by the approximation of $\ln\left( \frac{\kappa\cdot k_{B}}{h} \right)\approx ln (\frac{k_{B}}{h})$ and the assumption that the enthalpy of activation ${\Delta H}^{\ddagger}$and the entropy of activation ${\Delta S}^{\ddagger}$ are constant:

$\ln\left( \frac{k}{T} \right)vs.\frac{1}{T}$	**(4)**

**Figure S76**. Eyring Plot using rate constants derived by full line shape analysis of the VT NMR spectrum of **1**.

**Table S19.** Rate constants derived by full lineshape analysis.

| T [K] | 198 | 218 | 238 | 258 | 278 | 298 |
| --- | --- | --- | --- | --- | --- | --- |
| *k* [1/s] | $1.27 \cdot{10}^{1}$ | $2.12 \cdot{10}^{2}$ | $1.47 \cdot{10}^{3}$ | $7.98 \cdot{10}^{3}$ | $4.11 \cdot{10}^{4}$ | $8.86 \cdot{10}^{4}$ |

According to the linear Eyring plot, the enthalpy and entropy of activation were estimated to be ${\Delta H}^{\ddagger}$ = 41.9(14) kJ mol^−1^ (${\Delta H}^{\ddagger}$ = 10.0 kcal mol^−1^) and ${\Delta S}^{\ddagger}$ = −7.2(57) J K^−1^ mol^−1^ (${\Delta S}^{\ddagger}$ = −1.71 cal K^−1^ mol^−1^). Thereby, a Gibbs energy of activation of ${{\Delta G}^{\ddagger}}_{298K}$ = 44.0(14) kJ mol^−1^ (${{\Delta G}^{\ddagger}}_{298K}$ = 10.5 kcal mol^−1^) was derived.

# III. Mass Spectra

*exo,exo*-P_4_(PMes_2_)(O*^t^*Bu) **(4a)**

**Figure S77.** FD-MS spectrum (*n*-hexane) of *exo,exo*-P_4_(PMes_2_)(O*^t^*Bu) (**4a**).

**Figure S78.** Magnified FD-MS spectrum (*n*-hexane) of *exo,exo*-P_4_(PMes_2_)(O*^t^*Bu) (**4a**).

*exo,exo*-P_4_[P{N(*^i^*Pr)_2_}_2_](O*^t^*Bu) **(4b)**

**Figure S79.** ESI-MS spectrum (1,2-difluorobenzene) of *exo,exo*-P_4_[P{N(*^i^*Pr)_2_}_2_](O*^t^*Bu) (**4b**).

**Figure S80.** Magnified ESI-MS spectrum (1,2-difluorobenzene) of *exo,exo*-P_4_[P{N(*^i^*Pr)_2_}_2_](O*^t^*Bu) (**4b**).

*exo,exo*-P_4_[P{N(*^i^*Pr)_2_}_2_]{N(*^i^*Pr)_2_} **(4c)**

**Figure S81.** ESI-MS spectrum (1,2-difluorobenzene) of *exo,exo*-P_4_[P{N(*^i^*Pr)_2_}_2_]{N(*^i^*Pr)_2_} (**4c**).

**Figure S82.** Magnified ESI-MS spectrum (1,2-difluorobenzene) of *exo,exo*-P_4_[P{N(*^i^*Pr)_2_}_2_]{N(*^i^*Pr)_2_} (**4c**).

**Figure S83.** Formation of possible fragments observed in the MS-spectra of *exo,exo*-P_4_[P{N(*^i^*Pr)_2_}_2_]{N(*^i^*Pr)_2_} (**4c**).

*endo,exo*-P_4_(BPh_2_){N(*^i^*Pr)_2_} **(5)**

**Figure S84.** FD-MS spectrum (*n*-hexane) of P_4_(BPh_2_){N(*^i^*Pr)_2_} (**5**).

**Figure S85.** Magnified FD-MS spectrum (*n*-hexane) of P_4_(BPh_2_){N(*^i^*Pr)_2_} (**5**).

# IV. Single Crystal X-ray Diffraction Data

Single crystal X-ray diffraction data were recorded on an XtaLAB Synergy R DW diffractometer equipped with a HyPix-Arc 150 detector and using Cu-Kα (λ = 1.54184 Å) radiation. The single crystals were selected under mineral oil, mounted using a micro mount loop and frozen using an Oxford Cryosystems open flow N2 cooling device. Data were collected at 123.0(1) K and processed using the CrysAlisPro package.^[8]^ Using Olex2,^[9]^ the structures were subsequently solved using intrinsic phasing implemented in the structure solution program ShelXT^[10]^ and least-square refinements on *F^2^* were carried out with ShelXL^[11]^ refinement package. The hydrogen atoms were located in idealised positions and refined isotropically with a riding model. For compound (TMC)P–P{N(*i*Pr)_2_}_2_ the structural models were refined using non-spherical atomic form factors with NoSpherA2^[12]^ and the hydrogen atoms were refined anisotropically. Crystallographic data for the structures in this paper have been deposited with the Cambridge Crystallographic Data Centre, CCDC, 12 Union Road, Cambridge CB21EZ, UK. Copies of these data can be obtained free of charge (http://www.ccdc.cam.ac.uk) on quoting the deposition numbers 2491197–2491204, 2577702–2577704 and 2584727.

**Figure S86.** Solid-state molecular structure of (6Dipp)CuN(*^i^*Pr)_2_. Displacement ellipsoids are drawn at the 50 % probability level; H atoms are omitted for clarity. Selected bond lengths [Å] and angles [°]: Cu1–N3: 1.838(2), Cu1–C1:1.893(3), N1–C1: 1.347(3), N2–C1: 1.346(3), N1–C2: 1.471(3), N2–C4:1.473(3), N3–C29: 1.453(3), N3–C32: 1.452(4), C2–C3: 1.517(4), C3–C4: 1.515(4); N3–Cu1–C1: 178.4(2), N1–C1–N2: 116.3(2), C2–C3–C4: 109.3(2), C1–N2–C4: 126.6(2), C1–N1–C2: 124.0(2), N1–C2–C3: 109.7(2), N2–C4–C3: 110.7(2), C1–N1–C5: 120.4(2), C1–N2–C17: 119.1(2), Cu1–N3–C29: 124.26(17), Cu1–N3–C32: 123.09(18).

**Figure S87.** Solid-state molecular structure of **1**. Displacement ellipsoids are drawn at the 50 % probability level; H atoms are omitted for clarity. Selected bond lengths [Å] and angles [°]: Cu1−C1: 1.929(2), Cu1−P1: 2.3074(5), Cu1−P4: 2.3420(5), P1−P3: 2.1847(7), P1−P2: 2.1906(8), P2−P3: 2.1768(7), P2−P4: 2.2349(7), P3−P4: 2.2013(6), P4−O1: 1.641(1); P1−Cu1−P4: 72.32(2), Cu1−P1−P2: 94.38(2), Cu1−P1−P3: 100.85(2), Cu1−P4−P2: 92.28(2), Cu1−P4−P3: 99.29(2), Cu1−P4−O1: 140.12(5), P1−P2−P4: 76.61(2), P1−P2−P3: 60.03(2), P1−P3−P4: 77.44(2), P1−P3−P2: 60.30(2), P2−P1−P3: 59.67(2), P2−P4−P3: 58.76(2), P2−P4−O1: 117.50(6), P3−P4−O1: 118.34(5), P3−P2−P4: 59.85(2), P2−P3−P4: 61.39(2).

**Figure S88.** Solid-state molecular structure of **2**. Displacement ellipsoids are drawn at the 50 % probability level; H atoms are omitted for clarity. Selected bond lengths [Å] and angles [°]: Cu1−C1: 1.940(6), Cu1−P1: 2.296(2), Cu1−P4: 2.349(2), P1−P2: 2.209(3), P1−P3: 2.203(2), P3−P2: 2.184(3), P4−P2: 2.214(2), P4−P3: 2.218(2), P4−O1: 1.613(5), W1−P1: 2.585(2); P1−Cu1−P4: 71.69(6), Cu1−P1−P2: 97.80(8), Cu1−P1−P3: 99.84(8), Cu1−P4−P2: 96.13(8), Cu1−P4−P3: 97.84(8), Cu1−P4−O1: 140.0(2), Cu1−P1−W1: 144.07(8), P1−P2−P4: 75.92(8), P1−P2−P3: 60.21(8), P1−P3−P4: 75.95(8), P1−P3−P2: 60.46(8), P2−P1−P3: 59.34(8), P2−P4−P3: 59.05(8), P4−P2−P3: 60.57(8), P4−P3−P2: 60.38(8), P2−P4−O1: 117.5(2), P3−P4−O1: 117.3(2), P2−P1−W1: 111.11(8), P3−P1−W1: 112.93(8).

**Figure S89.** Solid-state molecular structure of **3**. Displacement ellipsoids are drawn at the 50 % probability level; H atoms are omitted for clarity. Selected bond lengths [Å] and angles [°]:Cu1−C1: 1.936(2), Cu1−P1: 2.3118(6), Cu1−P4: 2.3955(6), P1−P3: 2.190(1), P1−P2: 2.1924(9), P2−P3: 2.172(1), P2−P4: 2.2401(8), P3−P4: 2.2062(8), P4−N3: 1.706(2); P1−Cu1−P4: 73.79(2), Cu1−P1−P2: 99.71(3), Cu1−P1−P3: 93.12(3), Cu1−P4−P2: 95.91(3), Cu1−P4−P3: 90.45(3), Cu1−P4−N3: 145.76(7), P1−P2−P4: 79.25(3), P1−P2−P3: 60.23(3), P1−P3−P4: 80.05(3), P1−P3−P2: 60.35(3), P2−P1−P3: 59.42(3), P2−P4−P3: 58.47(3), P2−P4−N3: 116.43(7), P3−P4−N3: 115.27(7), P3−P2−P4: 59.98(3), P2−P3−P4: 61.55(3).

**Figure S90.** Solid-state molecular structure of **4b**. Displacement ellipsoids are drawn at the 50 % probability level; H atoms are omitted for clarity. Selected bond lengths [Å] and angles [°]: P1−P5: 2.2698(8), P4−O1: 1.6465(18), P1−P2: 2.2151(9), P1−P3: 2.2120(9), P2−P4: 2.2076(9), P2−P3: 2.1743(9), P3−P4: 2.2324(9), N1−P5: 1.684(2), N2−P5: 1.688(2); P1-P2-P4: 80.42(3), P1-P3-P4: 79.94(3), P2-P1-P3: 58.83(3), P2-P4-P3: 58.64(3), P2-P1-P5: 93.52(3), P3-P1-P5: 93.47(3), O1-P4-P2: 99.38(7), O1-P4-P3: 103.55(7), P1-P2-P3: 60.51(3), P3-P2-P4: 61.25(3), P1-P3-P2: 60.66(3), P2-P3-P4: 60.11(3).

**Figure S91.** Solid-state molecular structure of **4c**. Displacement ellipsoids are drawn at the 50 % probability level; H atoms are omitted for clarity. Selected bond lengths [Å] and angles [°]: P1−P5: 2.2605(5), P4−N1: 1.6919(13), P1−P2: 2.2083(6), P1−P3: 2.2171(6), P2−P4: 2.2510(6), P2−P3: 2.1952(6), P3−P4: 2.2053(6), N2−P5: 1.6896(13), N3−P5: 1.6892(13); P1-P2-P4: 79.63(2), P1-P3-P4: 80.44(2), P2-P1-P3: 59.478(19), P2-P4-P3: 59.014(19), P2-P1-P5: 94.11(2), P3-P1-P5: 92.87(2), N1-P4-P2: 108.29(5), N1-P4-P3: 106.31(5), P1-P2-P3: 60.459(19), P3-P2-P4: 59.453(18), P1-P3-P2: 60.063(18), P2-P3-P4: 61.533(19).

**Figure S92.** Solid-state molecular structure of **5**. Displacement ellipsoids are drawn at the 50 % probability level; H atoms are omitted for clarity. Selected bond lengths [Å] and angles [°]: B1−P1: 2.070(2), B1−P4: 1.983(2), P1−P2:2.2101(5), P1−P3:2.2052(5), P2−P4:2.1661(5), P2−P3:2.2288(6), P3−P4: 2.2096(5), P4−N1:1.649(1); P1-P2-P4: 73.16(2), P1-P3-P4: 72.42(2), P1-B1-P4: 80.07(6), P2-P1-P3: 60.63(2), P2-P4-P3: 61.23(2), N1-P4-B1: 130.59(6), N1-P4-P2: 122.63(5), N1-P4-P3: 126.07(5), B1-P1-P3: 95.33(5), B1-P1-P2: 92.66(5), B1-P4-P3: 97.73(5), B1-P4-P2: 96.47(5), P1-P2-P3: 59.58(2), P4-P2-P3: 60.35(2), P2-P3-P1: 59.79(2), P2-P3-P4: 58.42(2).

**Figure S93.** Solid-state molecular structure of **6**. Displacement ellipsoids are drawn at the 50 % probability level; H atoms are omitted for clarity. Selected bond lengths [Å] and angles [°]: P1−P2: 2.2273(8), P1−P3: 2.1875(8), P2−P3: 2.2260(7), P1−N1: 1.691(2), P2−P4: 2.1911(7), P3−P5: 2.2405(7), P4−C19: 1.773(2), P5−N2: 1.708(2), P5−N3: 1.701(2); P1-P2-P3: 58.84(2), P1‑P3‑P2: 60.61(3), P2-P1-P3: 60.55(2), P1-P2-P4: 97.91(3), P1-P3-P5: 99.09(3), N1-P1-P2: 109.46(9), P3-P2-P4: 104.05(3), P2‑P3-P5: 113.34(3), P2-P4-C19: 102.64(7).

**Figure S94.** Solid-state molecular structure of **7**. Displacement ellipsoids are drawn at the 50 % probability level; H atoms are omitted for clarity. Selected bond lengths [Å] and angles [°]: W1−P4: 2.534(1), P1−P5: 2.271(2), P4−N1: 1.678(4), P1−P2: 2.209(2), P1−P3: 2.207(2), P2−P4: 2.212(2), P2−P3: 2.194(2), P3−P4: 2.224(2); P1-P2-P4: 83.03(7), P1-P3-P4: 82.80(7), W1-P4-P2: 125.60(7), P2-P1-P3: 59.58(6), P2-P4-P3: 59.26(6), P2-P1-P5: 94.38(7), P1-P2-P3: 60.17(6), P4-P2-P3: 60.64(6), P2-P3-P1: 60.25(6), P2-P3-P4: 60.10(6).

**Figure S95.** Solid-state molecular structure of **8**. Displacement ellipsoids are drawn at the 50 % probability level; H atoms are omitted for clarity. Selected bond lengths [Å] and angles [°]: Cu1−P4: 2.2677(9), Cu1−P5: 2.222(1), Cu1−Br1: 2.2765(5), P1−P5: 2.257(1), P4−N1: 1.680(3), P1−P2: 2.175(1), P1−P3: 2.187(1), P2−P4: 2.232(1), P2−P3: 2.234(1), P3−P4: 2.206(1); P1-P2-P4: 89.59(4), P1-P3-P4: 89.96(4), P5-Cu1-P4: 96.38(3), P1-P5-Cu1: 105.27(4), Cu1-P4-P2: 114.76(4), P2-P1-P3: 61.60(4), P2-P4-P3: 60.43(4), P2-P1-P5: 106.15(5), P1-P2-P3: 59.46(4), P4-P2-P3: 59.21(4), P2-P3-P1: 58.94(4), P2-P3-P4: 60.36(4).

**Figure S96.** Solid-state molecular structure of (IPr)Cu(κ^2^-P_4_O*^t^*Bu). Displacement ellipsoids are drawn at the 50 % probability level; H atoms are omitted for clarity. Selected bond lengths [Å] and angles [°]: Cu1−C1: 1.919(2), Cu1−P1: 2.3066(6), Cu1−P4: 2.3575(5), P1−P3: 2.1976(8), P1−P2: 2.1954(8), P2−P3: 2.1726(9), P2−P4: 2.2154(8), P3−P4: 2.2197(8), P4−O1: 1.646(2); P1−Cu1−P4: 72.95(2), Cu1−P1−P2: 100.98(3), Cu1−P1−P3: 92.98(3), Cu1−P4−P2: 98.83(2), Cu1−P4−P3: 91.05(2), Cu1−P4−O1: 142.42(6), P1−P2−P4: 77.91(3), P1−P2−P3: 60.41(3), P1−P3−P4: 77.77(3), P1−P3−P2: 60.31(3), P2−P1−P3: 59.28(3), P2−P4−P3: 58.66(3), P2−P4−O1: 118.00(6), P3−P4−O1: 113.56(7), P3−P2−P4: 60.77(3), P2−P3−P4: 60.57(2).

**Figure S97.** Solid-state molecular structure of (TMC)P–P{N(*i*Pr)_2_}_2_. Displacement ellipsoids are drawn at the 50 % probability level; H atoms are omitted for clarity. Selected bond lengths [Å] and angles [°]: P1−P2: 2.2041(2), P1−N1: 1.7102(6), P1−N2: 1.7121(6), P2−C13: 1.7984(7), N3−C13: 1.3648(9), N4−C13: 1.3632(8), C14−C15: 1.361(1), N1−C1: 1.4682(9), N1−C4: 1.4729(9), N2−C7: 1.4782(9), N2−C10: 1.4677(9); P1-P2-C13: 98.96(2), P2-P1-N2: 99.12(2), P2-P1-N1: 103.31(2), N3-C13-N4: 104.65(6), N1-P1-N2: 109.60(3).

**Table 20.** Crystallographic data and refinement details.

| **Compound** | **(6Dipp)CuN(*^i^*Pr)_2_** | **1** | **2** | **3** | **4b** | **4c** |
| --- | --- | --- | --- | --- | --- | --- |
| Empirical formula | C_34_H_54_CuN_3_ | C_32_H_49_CuN_2_OP_4_ | C_37_H_49_CuN_2_O_6_P_4_W | C_34_H_54_CuN_3_P_4_ | C_16_H_37_N_2_OP_5_ | C_18_H_42_N_3_P_5_ |
| Formula weight | 568.34 | 665.15 | 989.05 | 692.22 | 428.32 | 455.39 |
| Temperature/K | 123.0(1) | 123.0(1) | 123.0(1) | 123.0(1) | 123.0(1) | 123.0(1) |
| Crystal system | orthorhombic | triclinic | orthorhombic | triclinic | monoclinic | monoclinic |
| Space group | Pna2_1_ | *P*-1 | *P*2_1_2_1_2_1_ | P-1 | P2_1_/c | P2_1_/c |
| a/Å | 17.5482(1) | 10.1523(2) | 10.7134(1) | 10.3584(2) | 12.4262(3) | 7.6297(1) |
| b/Å | 17.5187(1) | 10.8947(2) | 18.3473(2) | 10.8909(3) | 21.6621(4) | 27.1573(4) |
| c/Å | 10.6008(1) | 16.6044(3) | 21.5379(3) | 17.4891(4) | 9.0087(2) | 12.3723(2) |
| α/° | 90 | 79.409(1) | 90 | 97.285(2) | 90 | 90 |
| β/° | 90 | 88.314(1) | 90 | 90.110(2) | 99.696(2) | 93.2360(10) |
| γ/° | 90 | 75.5230(10) | 90 | 108.274(2) | 90 | 90 |
| Volume/Å^3^ | 3258.92(4) | 1747.68(6) | 4233.53(8) | 1856.54(8) | 2390.32(9) | 2559.48(7) |
| Z | 4 | 2 | 4 | 2 | 4 | 4 |
| ρ_calc_g/cm^3^ | 1.158 | 1.264 | 1.552 | 1.238 | 1.190 | 1.182 |
| μ/mm^‑1^ | 1.115 | 2.809 | 7.353 | 2.650 | 3.600 | 3.370 |
| F(000) | 1232.0 | 704.0 | 1984.0 | 736.0 | 920.0 | 984.0 |
| Crystal size/mm^3^ | 0.72 × 0.469 × 0.408 | 0.18 × 0.12 × 0.04 | 0.252 × 0.16 × 0.091 | 0.249 × 0.201 × 0.187 | 0.533 × 0.309 × 0.205 | 0.088 × 0.064 × 0.06 |
| Radiation | Cu Kα (λ = 1.54184) | Cu Kα (λ = 1.54184) | Cu Kα (λ = 1.54184) | Cu Kα (λ = 1.54184) | CuKα (λ = 1.54184) | Cu Kα (λ = 1.54184) |
| 2Θ range for data collection/° | 7.13 to 133.81 | 5.416 to 150.322 | 8.21 to 133.026 | 8.628 to 133.534 | 7.218 to 133.806 | 6.51 to 148.5 |
| Index ranges | -20 ≤ h ≤ 20, -20 ≤ k ≤ 20, -12 ≤ l ≤ 10 | -12 ≤ h ≤ 12, -13 ≤ k ≤ 13, -18 ≤ l ≤ 20 | -9 ≤ h ≤ 12, -21 ≤ k ≤ 21, -25 ≤ l ≤ 23 | -12 ≤ h ≤ 12, -12 ≤ k ≤ 12, -14 ≤ l ≤ 20 | -14 ≤ h ≤ 14, -25 ≤ k ≤ 25, -10 ≤ l ≤ 8 | -9 ≤ h ≤ 9, -33 ≤ k ≤ 33, -15 ≤ l ≤ 15 |
| Reflections collected | 31558 | 29909 | 22508 | 26004 | 32557 | 46358 |
| Independent reflections | 4959 [R_int_ = 0.0511, R_sigma_ = 0.0231] | 7060 [R_int_ = 0.0352, R_sigma_ = 0.0270] | 7219 [R_int_ = 0.0532, R_sigma_ = 0.0482] | 6560 [R_int_ = 0.0549, R_sigma_ = 0.0347] | 4250 [R_int_ = 0.0806, R_sigma_ = 0.0325] | 5203 [R_int_ = 0.0389, R_sigma_ = 0.0176] |
| Data/restraints/parameters | 4959/1/355 | 7060/0/372 | 7219/6/471 | 6560/0/391 | 4250/0/228 | 5203/0/247 |
| Goodness-of-fit on F^2^ | 1.053 | 1.041 | 1.035 | 1.020 | 1.083 | 1.034 |
| Final R1, wR2 [I>=2σ (I)] | 0.0366, 0.1030 | 0.0341, 0.0911 | 0.0340, 0.0877 | 0.0417, 0.1089 | 0.0511, 0.1390 | 0.0317, 0.0842 |
| Final R1, wR2 [all data] | 0.0368, 0.1033 | 0.0374, 0.0928 | 0.0354, 0.0891 | 0.0446, 0.1116 | 0.0538, 0.1419 | 0.0342, 0.0858 |
| Largest diff. peak/hole / e Å^-3^ | 0.27/-0.66 | 0.55/-0.49 | 1.49/-0.78 | 0.71/-0.48 | 0.51/-0.53 | 0.57/-0.30 |
| Flack parameter | 0.00(3) | - | -0.026(6) | - | - | - |

**Table 21.** Crystallographic data and refinement details.

| **Compound** | **5** | **6** | **7** | **8** | **[(IPr)Cu(κ^2^-P_4_O*^t^*Bu)]** | **(TMC)P–P{N(*i*Pr)_2_}_2_** |
| --- | --- | --- | --- | --- | --- | --- |
| Empirical formula | C_18_H_24_BNP_4_ | C_44_H_78_N_5_P_5_ | C_23_H_42_N_3_O_5_P_5_W | C_18_H_42_BrCuN_3_P_5_ | C_31_H_45_CuN_2_OP_4_ | C_19_H_40_N_4_P_2_ |
| Formula weight | 389.07 | 831.96 | 779.29 | 598.84 | 649.11 | 386.504 |
| Temperature/K | 123.0(1) | 123.0(1) | 123.0(1) | 123.0(1) | 123.0(1) | 123.00(10) |
| Crystal system | triclinic | monoclinic | orthorhombic | monoclinic | triclinic | monoclinic |
| Space group | P-1 | P2_1_/n | Pbca | P2_1_/n | P-1 | P2_1_/n |
| a/Å | 8.4590(3) | 18.5347(1) | 20.3425(4) | 15.5353(3) | 10.0729(3) | 8.0199(1) |
| b/Å | 9.2103(3) | 14.4203(1) | 12.6341(2) | 24.9307(4) | 10.5848(3) | 25.9087(2) |
| c/Å | 13.5903(4) | 19.2281(1) | 25.6912(7) | 15.7480(3) | 16.6197(3) | 11.5023(1) |
| α/° | 80.678(3) | 90 | 90 | 90 | 79.460(2) | 90 |
| β/° | 87.430(3) | 101.9170(10) | 90 | 108.943(2) | 86.894(2) | 103.896(1) |
| γ/° | 73.452(3) | 90 | 90 | 90 | 73.309(2) | 90 |
| Volume/Å^3^ | 1001.56(6) | 5028.45(5) | 6602.9(2) | 5768.97(19) | 1668.69(8) | 2320.06(4) |
| Z | 2 | 4 | 8 | 8 | 2 | 4 |
| ρ_calc_g/cm^3^ | 1.290 | 1.099 | 1.568 | 1.379 | 1.292 | 1.107 |
| μ/mm^‑1^ | 3.468 | 1.931 | 3.776 | 5.385 | 2.931 | 1.758 |
| F(000) | 408.0 | 1808.0 | 3120.0 | 2480.0 | 684.0 | 852.5 |
| Crystal size/mm^3^ | 0.136 × 0.097 × 0.045 | 0.139 × 0.1 × 0.077 | 0.18 × 0.12 × 0.04 | 0.17 × 0.1 × 0.05 | 0.309 × 0.17 × 0.098 | 0.26 × 0.18 × 0.13 |
| Radiation | Cu Kα (λ = 1.54184) | Cu Kα (λ = 1.54184) | Mo Kα (λ = 0.71073) | Cu Kα (λ = 1.54184) | Cu Kα (λ = 1.54184) | Cu Kα (λ = 1.54184) |
| 2Θ range for data collection/° | 6.59 to 145.674 | 6.03 to 150.236 | 4.004 to 52.74 | 6.912 to 152.386 | 8.858 to 133.52 | 6.82 to 150.32 |
| Index ranges | -9 ≤ h ≤ 10, -11 ≤ k ≤ 10, -16 ≤ l ≤ 15 | -23 ≤ h ≤ 23, -17 ≤ k ≤ 17, -23 ≤ l ≤ 23 | -25 ≤ h ≤ 25, -15 ≤ k ≤ 14, -32 ≤ l ≤ 32 | -19 ≤ h ≤ 19, -31 ≤ k ≤ 31, -19 ≤ l ≤ 19 | -11 ≤ h ≤ 11, -12 ≤ k ≤ 12, -15 ≤ l ≤ 19 | -9 ≤ h ≤ 9, -32 ≤ k ≤ 31, -13 ≤ l ≤ 14 |
| Reflections collected | 10985 | 65961 | 79833 | 298754 | 23000 | 30778 |
| Independent reflections | 3794 [R_int_ = 0.0190, R_sigma_ = 0.0240] | 10239 [R_int_ = 0.0359, R_sigma_ = 0.0269] | 6735 [R_int_ = 0.0674, R_sigma_ = 0.0261] | 11897 [R_int_ = 0.0829, R_sigma_ = 0.0210] | 5882 [R_int_ = 0.0449, R_sigma_ = 0.0326] | 4716 [R_int_ = 0.0322, R_sigma_ = 0.0191] |
| Data/restraints/parameters | 3794/0/221 | 10239/61/544 | 6735/0/388 | 11897/14/549 | 5882/0/373 | 4716/0/478 |
| Goodness-of-fit on F^2^ | 1.053 | 1.031 | 1.278 | 1.079 | 1.102 | 1.041 |
| Final R1, wR2 [I>=2σ (I)] | 0.0253, 0.0667 | 0.0545, 0.1574 | 0.0381, 0.0821 | 0.0552, 0.1604 | 0.0323, 0.0950 | 0.0193, 0.0529 |
| Final R1, wR2 [all data] | 0.0291, 0.0683 | 0.0614, 0.1643 | 0.0482, 0.0853 | 0.1262, 0.2849 | 0.0347, 0.0972 | 0.0215, 0.0537 |
| Largest diff. peak/hole / e Å^-3^ | 0.32/-0.20 | 0.70/-0.38 | 1.78/-0.80 | 1.69/-1.34 | 0.44/-0.45 | 0.20/-0.17 |
| Flack parameter | - | - | - | - | - | - |

# V. DFT Calculations

**Computational Details**

Geometry optimisation and frequency analysis were carried out with the ORCA program version 5.0.3^[13]^ using the composite method r2scan-3c with the dielectric constant of THF.^[14]^ The geometry for [(6Dipp)Cu(κ^2^-P_4_O*^t^*Bu)] (**1**) was initially optimised. The transition state for the dynamic process observed by ^31^P{^1^H} NMR spectroscopy was determined by the Nudged Elastic Band Method^[15]^ followed by an additional transition state optimisation step. The optimised transition state shows one negative frequency corresponding to the tumbling of the P_4_ unit. The thermodynamic parameters of this process are: ΔG^0^ = 9.84 kcal mol^−1^, ΔH^0^ = 8.97 kcal mol^−1^ and ΔS^0^ = −0.88 cal K^−1^ mol^−1^ at 298.15 K and 1 atm.

**Figure S98.** Optimised structure of the transition state.

**Cartesian coordinates of the transition state structure of [(6Dipp)Cu(κ^2^‑P_4_O*^t^*Bu)]**

Cu 0.98856494005249	0.04474062486876	0.46308791499057

P	3.47447197447828	1.02209181904065	1.46773390819157

P	2.81298885505863	-0.85140080327368	-0.56038532360523

P	4.33427450168006	-0.96631865364416	1.04066195182677

P	2.42530088747698	-0.75600820014902	2.11000167997067

O	2.86377981308359	-1.97682629103184	-1.78401467565476

N	-1.57969279404238	1.01250246572238	1.38716555460588

N	-1.38284901551352	0.97389230773656	-0.90161609779457

C	-0.83026463940715	0.75556486851805	0.30272060101290

C	-1.02436578203260	0.75877419398739	2.68849371161319

C	-0.63083599931945	0.62997612292127	-2.07660175983230

C	0.16274054695206	1.61468651214414	-2.68637466687841

C	-1.11073897533850	-0.53454481792493	3.22986016496824

C	-2.96759004123290	1.50849780298332	1.34098464716550

H	-3.13586953601647	2.11854162860107	2.23494910517004

H	-3.66250499514128	0.65651379457369	1.38194537881758

C	0.25265349523877	3.02855546174259	-2.14454468768831

H	-0.39084226665150	3.09686880173675	-1.26015117996652

C	-1.81110210286535	-1.67042543581698	2.50754277684718

H	-2.04076145171593	-1.33805127814166	1.48896493743902

C	-0.44324562479665	1.82785839528399	3.38976337137463

C	0.88574641198695	1.25433874260510	-3.82522101818147

H	1.52289233390907	1.98878187452805	-4.31096113938114

C	0.80371608447404	-0.03208562085935	-4.34507475520152

H	1.37751217206061	-0.29609978894549	-5.22943268946501

C	-0.74527676969636	-0.66999923386082	-2.59423765917361

C	-2.74138288130515	1.50071793630170	-1.12540243781045

H	-3.43327523838244	0.66430322652023	-1.30446228608744

H	-2.71644252923354	2.11313641254679	-2.03349501811937

C	-0.41871928800860	3.23882918582079	2.83273086809473

H	-0.95626105558520	3.23959101945878	1.87885957016203

C	3.51198355466550	-3.29766946665905	-1.82058872281697

C	-3.18095472386780	2.31898193869853	0.07485692957951

H	-2.59805338774227	3.24691302067029	0.12358340878110

H	-4.23661047033567	2.58768848301897	-0.02354036775295

C	-0.00953757341943	-0.98176490043433	-3.73945391760534

H	-0.06501347649969	-1.98410752844658	-4.15624320154508

C	3.08131204243286	-4.10970513297548	-0.60447012960883

H	3.43687886408877	-3.65455662942074	0.32633443498773

H	3.49913156066652	-5.11966212333057	-0.66960086405391

H	1.98962499560903	-4.18558469527051	-0.56438180194768

C	5.02395590544584	-3.11342415975286	-1.87686551884812

H	5.30018634419955	-2.49406712745278	-2.73612500113165

H	5.50965672283192	-4.08947291351831	-1.97913066679651

H	5.40260233434921	-2.64166534457167	-0.96517130837824

C	0.11266931712042	1.56207616364717	4.64272304213626

H	0.58891087905341	2.36480103469738	5.19950235900477

C	3.00034910096875	-3.93321669576047	-3.10848801534618

H	1.91216877054783	-4.04421332283943	-3.07444108883013

H	3.44895297840182	-4.92321979621082	-3.24054659212737

H	3.26414487761006	-3.31212156522567	-3.97045241134317

C	-3.13815388734739	-2.00497846442088	3.20706032484090

H	-3.67879438850736	-2.77791758291272	2.64918927501881

H	-3.77970866223607	-1.12055061413601	3.28454930957461

H	-2.95610987776634	-2.38120306641032	4.22041667045740

C	-0.55695477685803	-0.74787306009321	4.49405232594455

H	-0.60370630147940	-1.73760031619742	4.94023994870551

C	-1.62956840194341	-1.72200180925711	-1.95208393268147

H	-2.11647195240490	-1.27653481378925	-1.07802219967320

C	-2.73127709040982	-2.18172648283166	-2.91718816919202

H	-2.30458208604158	-2.68728247241128	-3.79078302166964

H	-3.32494768943916	-1.33228371688607	-3.27220101093306

H	-3.40274236105772	-2.88693782775024	-2.41484306446879

C	-1.13535630261157	4.21687688660555	3.77492409484545

H	-2.15653417464436	3.88378384581381	3.99016879845711

H	-1.18540841281529	5.21118793611315	3.31777316430673

H	-0.60149148353910	4.31132978469169	4.72682489590122

C	0.05972824977416	0.28529600958976	5.18858088038065

H	0.49706135915183	0.09540315695167	6.16511714134079

C	1.68066966825894	3.36660577020093	-1.69601117248345

H	2.37672449727708	3.34482779700782	-2.54244473858161

H	2.03447306894001	2.65135610003595	-0.94542523682483

H	1.71087396597377	4.37133578435498	-1.25922675798732

C	-0.25553867822250	4.04794730379023	-3.17402968869601

H	-0.25733228475367	5.05378652131757	-2.73961291127946

H	-1.27457899616657	3.81024785116003	-3.49819012092767

H	0.38760348822999	4.06622443332521	-4.06105809883356

C	-0.92955615939448	-2.91972803367545	2.39145494897500

H	-0.69972032181113	-3.34157484824646	3.37596347255275

H	0.01465745303439	-2.68584866282043	1.88852311041255

H	-1.44843503509374	-3.68961640145370	1.81014354414677

C	1.01225943632415	3.70597860559889	2.54031039020125

H	1.61901594159427	3.72283673671807	3.45290419734855

H	1.00185690434877	4.71774989499483	2.11930084873450

H	1.49800063952497	3.03737854788287	1.82175319641818

C	-0.80455274474417	-2.91341146324134	-1.45053764221646

H	-1.45248373277816	-3.63474320980213	-0.93990175436330

H	-0.03215720695133	-2.58202017552060	-0.74687536005175

H	-0.31174130970822	-3.42933225715390	-2.28180697146732

FINAL SINGLE POINT ENERGY	-4439.294079352061 Hartree

**Cartesian coordinates of the optimised geometry of [(6Dipp)Cu(κ^2^‑P_4_O*^t^*Bu)]**

Cu 5.17674508826042	4.40400724328516	4.81869572622730

P	8.56018771830528	3.94173596748380	4.64461520917814

P	6.72153147456326	2.85947372995173	3.93010229921568

P	7.70396455975976	2.35215620671365	5.90945108517862

P	7.05766935998611	4.45011253872933	6.19668949132051

O	6.82373482239962	1.77378180864463	2.66383629775644

N	2.60948776907264	5.47923566205347	5.49083713171497

N	2.92252828352066	5.29456211951173	3.22131926220268

C	3.39848573340082	5.12130490635734	4.46480135987026

C	3.09618204841588	5.26323933704049	6.82599822710073

C	3.72398086789031	4.85325133477662	2.11291165637044

C	4.71261886340563	5.70921298260281	1.60126508122644

C	3.02339205570112	3.97735834303692	7.38560191378383

C	1.24652419258632	6.01544206302795	5.33559423725295

H	1.03380336061924	6.65650644486254	6.19773908088707

H	0.52181163446201	5.18708750519648	5.34684638329975

C	4.98610802981811	7.07968376167393	2.19185757547190

H	4.28670612090232	7.24159314889510	3.01977670787152

C	2.42690146457914	2.79460808177108	6.64542267625055

H	2.16312048072200	3.11860045926794	5.63227564016265

C	3.63361164353122	6.35536841331673	7.52813205299037

C	5.46369793573563	5.26046612305479	0.51213090456019

H	6.24392551422254	5.89623859742961	0.10170848082160

C	5.22315564511286	4.01615762669279	-0.05771416213832

H	5.81362173085766	3.68625826012282	-0.90837126613734

C	3.46806721398367	3.58934097972792	1.55779519037463

C	1.63187593708914	5.92415597710124	2.88377813478179

H	0.89032292144955	5.14539863584687	2.65318020077537

H	1.77994580092217	6.52016350329941	1.97591067130476

C	3.75319532598544	7.72984855347351	6.89795560317995

H	3.17466644614598	7.73292222234566	5.96873472881089

C	7.98499651706676	1.18532965180832	1.98374856231798

C	1.15911563471167	6.79583456806116	4.03438657039654

H	1.78473127255389	7.69434051994644	4.10041739182819

H	0.12830997930901	7.11562017394360	3.85657707290366

C	4.23324170727676	3.19051203728580	0.45974010547352

H	4.06289244651337	2.21388288432379	0.01420931724962

C	8.87407918551269	0.47160319709459	2.99484705504061

H	9.34035505606141	1.18132530929936	3.68625777565640

H	9.67309224719259	-0.06259984661466	2.46985677526748

H	8.29250380636280	-0.25419279656786	3.57271051874432

C	8.74030137726678	2.28513058613387	1.24623854776851

H	8.07014345606641	2.80784052598345	0.55609281755689

H	9.56401841901482	1.84724211784700	0.67241758109908

H	9.16320590974385	3.01353292440099	1.94533225563079

C	4.12567907194057	6.12999056184706	8.81420183598610

H	4.56962349644445	6.95065951757677	9.37112023234592

C	7.37794778242882	0.19291428822357	0.99799706588314

H	6.80605539877639	-0.57410792565242	1.52996656665479

H	8.17098045804001	-0.29670654715923	0.42328440141624

H	6.71011022757915	0.71046027026768	0.30146649777404

C	1.14161069742078	2.31156274851335	7.33334181765256

H	0.68709187674052	1.49788796061124	6.75715382229010

H	0.41164215139916	3.12352726659267	7.41996150985893

H	1.35350153492541	1.93572038435041	8.34073094129044

C	3.52411656642814	3.80139648985496	8.67883610391706

H	3.48752271251407	2.81714054803867	9.13810577902118

C	2.42000438322944	2.65130603327693	2.12630894507553

H	1.94419677689481	3.14289649332656	2.98199571989823

C	1.32757453900242	2.34359118719229	1.09276405247101

H	1.73882955816172	1.79794303130248	0.23604968831794

H	0.86639496627145	3.26410996403388	0.71873035028796

H	0.54457697687606	1.72280244940751	1.54225594200107

C	3.19226838412728	8.83571169245864	7.79996561902975

H	2.15684830673435	8.62449142690092	8.08832826876392

H	3.21447297812411	9.79535241894456	7.27215268258964

H	3.78482175909739	8.94637043469859	8.71426530573095

C	4.07251995588599	4.86374108584618	9.38528919322763

H	4.46418247501429	4.70388354210671	10.38625791394969

C	6.40763901236677	7.15788205219512	2.76574916632241

H	7.15900366557526	7.02364840861435	1.97910871739058

H	6.56517842077624	6.38122153489069	3.52510533723697

H	6.57559253768364	8.13461687995511	3.23330040915800

C	4.75107377629517	8.18753542961654	1.15504464079175

H	4.89053712837321	9.17154082723140	1.61623046067963

H	3.73564522363900	8.13821870229787	0.74726488581207

H	5.45700264963173	8.10306662966748	0.32148652782249

C	3.44125897655494	1.65100084163562	6.51098935416837

H	3.72304081409998	1.25107065169103	7.49128041976826

H	4.35275884280730	1.99672502854372	6.00786120402088

H	3.01312605968501	0.83236890051721	5.92232265424061

C	5.21339216373621	8.01294486277135	6.51578077290677

H	5.85137746572133	8.03823031024226	7.40694168431394

H	5.29451631416273	8.97984350025618	6.00603779212312

H	5.59497589602252	7.23223069052513	5.84806676305703

C	3.06093405778415	1.35898348136329	2.65136407421853

H	2.29792394357752	0.71881864807302	3.10854648992300

H	3.82298073837725	1.58190466093884	3.40530555657426

H	3.53714492601308	0.79462535517305	1.84162419743675

FINAL SINGLE POINT ENERGY	-4439.309029471462 Hartree

# VI.  References

[1]	M. Iglesias, D. J. Beetstra, J. C. Knight, L.-L. Ooi, A. Stasch, S. Coles, L. Male, M. B. Hursthouse, K. J. Cavell, A. Dervisi et al., *Organometallics* **2008**, *27*, 3279.

[2]	A. J. Jordan, C. M. Wyss, J. Bacsa, J. P. Sadighi, *Organometallics* **2016**, *35*, 613.

[3]	R. A. Bartlett, M. M. Olmstead, P. P. Power, G. A. Sigel, *Inorg. Chem.* **1987**, *26*, 1941.

[4]	J. Österlof, *Acta Chem. Scand.* **1950**, *4*, 374.

[5]	Peter H.M. Budzelaar, *gNMR*. *NMR Simulation Program*, IvorySoft, **2006**.

[6]	a) S. Aime, R. K. Harris, E. M. McVicker, M. Fild, *J. Chem. Soc., Dalton Trans.* **1976**, 2144; b) J. P. Albrand, H. Faucher, D. Gagnaire, J. B. Robert, *Chem. Phys. Lett.* **1976**, *38*, 521; c) M. A. M. Forgeron, M. Gee, R. E. Wasylishen, *J. Phys. Chem. A* **2004**, *108*, 4895; d) E. G. Finer, R. K. Harris, *Mol. Phys.* **1967**, *13*, 65; e) H. C. E. McFarlane, W. McFarlane, J. A. Nash, *J. Chem. Soc., Dalton Trans.* **1980**, 240.

[7]	C. L. B. Macdonald, J. F. Binder, A. Swidan, J. H. Nguyen, S. C. Kosnik, B. D. Ellis, *Inorg. Chem.* **2016**; 55, 14, 7152–7166.

[8]	*CrysAlisPro*, Rigaku Oxford Diffraction, Oxford, UK, **2020**.

[9]	O. V. Dolomanov, L. J. Bourhis, R. J. Gildea, J. A. K. Howard, H. Puschmann, *J. Appl. Crystallogr.* **2009**, *42*, 339.

[10]	G. M. Sheldrick, *Acta Crystallogr. A* **2015**, *71*, 3.

[11]	G. M. Sheldrick, *Acta Crystallogr. C* **2015**, *71*, 3.

[12]	F. Kleemiss, O. V. Dolomanov, M. Bodensteiner, N. Peyerimhoff, L. Midgley, L. J. Bourhis, A. Genoni, L. A. Malaspina, D. Jayatilaka, J. L. Spencer et al., *Chem. Sci.* **2020**, 12, 1675.

[13]	a) F. Neese, *WIREs Comput. Mol. Sci.* **2012**, *2*, 73; b) F. Neese, *WIREs Comput. Mol. Sci.* **2018**, *8*, e1327.

[14]	S. Grimme, A. Hansen, S. Ehlert, J.-M. Mewes, *J. Chem. Phys.* **2021**, *154*, 64103.

[15]	V. Ásgeirsson, B. O. Birgisson, R. Bjornsson, U. Becker, F. Neese, C. Riplinger, H. Jónsson, *J. Chem. Theory Comput.* **2021**, *17*, 4929.
